# Quail-chick grafting experiments corroborate that Tbr1-positive eminential prethalamic neurons migrate along three streams into hypothalamus, subpallium and septocommissural areas

**DOI:** 10.1007/s00429-020-02206-3

**Published:** 2021-02-05

**Authors:** Antonia Alonso, Carmen María Trujillo, Luis Puelles

**Affiliations:** 1 Department of Human Anatomy and Psychobiology, Faculty of Medicine, School of Medicine, University of Murcia, 30100 Murcia, Spain; 2 Biomedical Research Laboratory (LAIB), Health Campus, Murcia Biomedical Research Institute (IMIB-Arrixaca), El Palmar, 30120 Murcia, Spain; 3 Department of Biochemistry, Microbiology, Cell Biology and Genetics, Faculty of Sciences, School of Biology, University of La Laguna, 38200 La Laguna, Canary Islands Spain

**Keywords:** Prethalamic eminence, Quail-chick chimeras, Tbr1, Commissural septum, Lateral hypothalamus, Preoptic area, Diagonal band, Extended amygdala

## Abstract

The prethalamic eminence (PThE), a diencephalic caudal neighbor of the telencephalon and alar hypothalamus, is frequently described in mammals and birds as a transient embryonic structure, undetectable in the adult brain. Based on descriptive developmental analysis of *Tbr1* gene brain expression in chick embryos, we previously reported that three migratory cellular streams exit the PThE rostralward, targeting multiple sites in the hypothalamus, subpallium and septocommissural area, where eminential cells form distinct nuclei or disperse populations. These conclusions needed experimental corroboration. In this work, we used the homotopic quail-chick chimeric grafting procedure at stages HH10/HH11 to demonstrate by fate-mapping the three predicted tangential migration streams. Some chimeric brains were processed for *Tbr1 *in situ hybridization, for correlation with our previous approach. Evidence supporting all three postulated migration streams is presented. The results suggested a slight heterochrony among the juxtapeduncular (first), the peripeduncular (next), and the eminentio-septal (last) streams, each of which followed differential routes. A possible effect of such heterochrony on the differential selection of medial to lateral habenular hodologic targets by the migrated neurons is discussed.

## Introduction

**Fig. 1:**
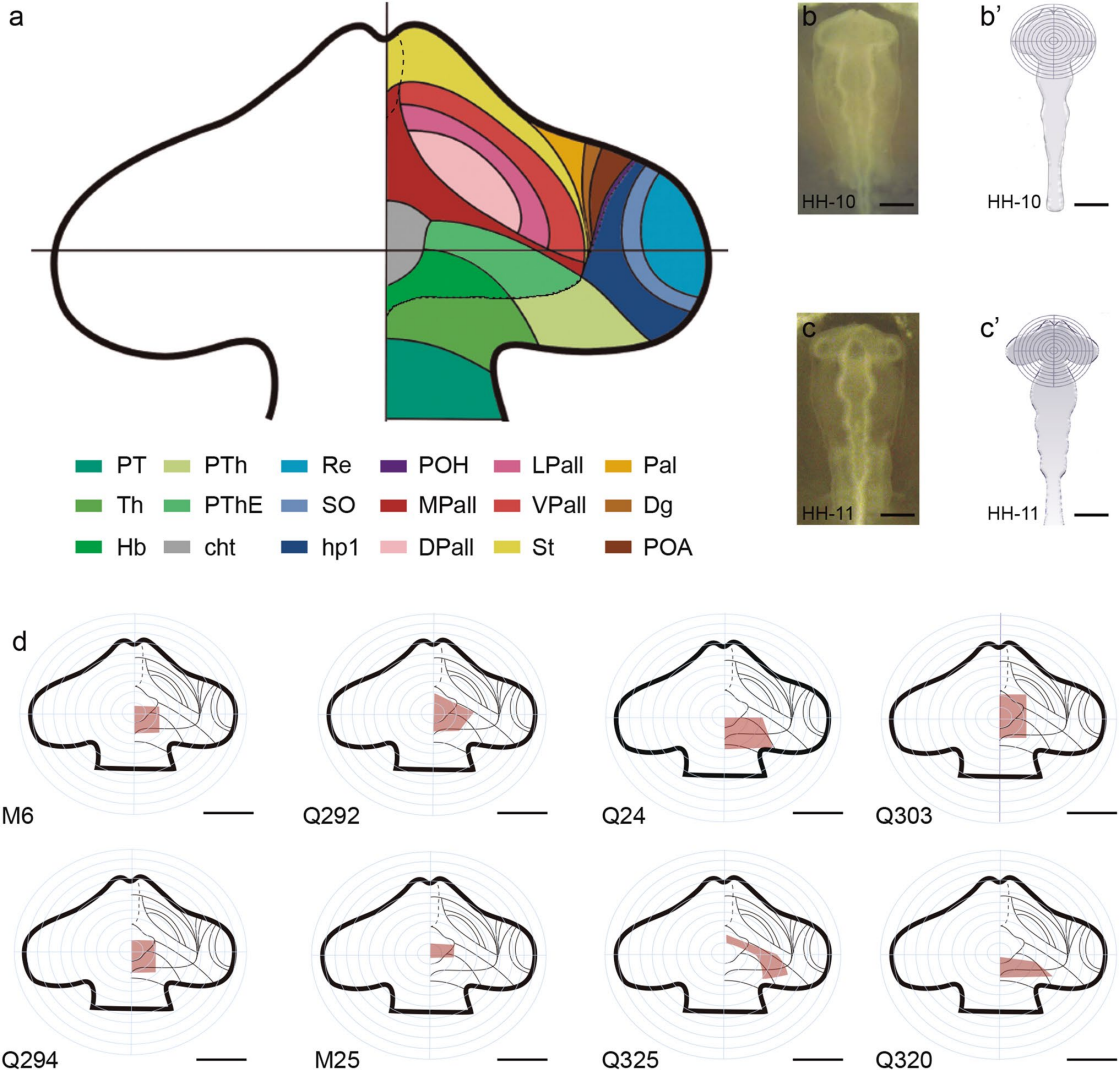
Schematic representations of the main types of grafts performed in the present study. **a** Consensus fate map of the embryonic chick prosencephalon at HH10 stage (data from Trujillo and Alvarado-Mallart 1991; García-López et al. 2004; Pombero and Martínez 2009). **b** Photomicrograph of a chicken embryo at HH10. **b’** Schematic drawing of **b** showing the placement of the ocular graticule used to normalize the operations. **c** Photomicrograph of a chicken embryo at HH11. **c’** Schematic drawing of **c** representing the placement of the ocular graticule used to normalize the operations. **d** Schematic drawings of the cases described in the present study, representing the place where the orthotopic and isochronic graft was made relative to the fate map of the presumptive prosencephalon. Scale bar in **b–b’**, 400 μm, **d**, 250 μm

The prethalamic eminence (PThE) is a firm candidate to produce excitatory neurons invading neighboring septocommissural, subpallial and hypothalamic histogenetic units (Abellán et al. 2010a; Bupesh et al. 2011; Medina and Abellán 2012; Watanabe et al. 2018; Alonso et al. 2020). It represents a molecularly distinct diencephalic progenitor subdomain found at the dorsalmost alar plate portion of prosomere 3 (p3; prethalamus); the rostral border of the PThE contacts directly the telencephalon, as well as the alar hypothalamic paraventricular domain of the hp1 prosomere (see Fig. 1a; Puelles et al., 2000,2012a; b; Puelles and Rubenstein 2003, 2015; Ferrán et al. 2015b). It was postulated recently that during early telencephalic evagination a rostrodorsal part of the PThE (carrying prospective chorioidal tissue of its adjacent roof plate) co-evaginates into the caudomedial wall of the nascent hemisphere, contributing there to the formation of the ulterior chorioidal fissure (Puelles 2019). Characteristic developmental gene markers of the PThE include *Calb2*, *Lhx9*, *Lhx5*, *VGlut2, Tbr1*, *Tbr2* and *Pax6* (the latter is expressed selectively in ventricular PThE cells, similarly as occurs in the telencephalic pallium) (see other markers in Bulfone et al. 1995, 1999; Abbott and Jacobowitz 1999; Rétaux et al. 1999; Puelles et al. 2000; Abellán et al. 2010b; Adutwum-Ofosu et al. 2016; Ruiz-Reig and Studer 2017; Ruiz-Reig et al. 2017). This molecular profile is consistent with a progenitor population producing glutamatergic neurons (Englund et al. 2005; Hevner et al. 2006). The glutamatergic identity of eminential neurons distinguishes the PThE from other more ventral alar prethalamic microzones, where the activity of master genes such as *Dlx2, Arx1* and *Pax6* (in the mantle) lead to GABAergic neuronal phenotypes (Puelles et al. 2000; Shimogori et al. 2010).

PThE depopulation. As a consequence of its partial evagination, the eminential ventricular zone forms a bent bridge between the 3rd ventricle and the lateral ventricle, so that its ventromedial part bulges markedly into the caudal aspect of the interventricular foramen (Puelles et al. 2012b). The eminential bulge was classically wrongly ascribed to the thalamus (the now outdated synonymous term ‘eminentia thalami’ is still found in the literature; see TNA [Terminologia Neuroanatomica] 2017). The mouse PThE is clearly visible at embryonic stages (Puelles et al. 2000) but tends to become cryptic at perinatal or postnatal stages, though PThE remnants can be identified in the mouse with specific molecular markers (Puelles et al. 2020). This evanescent pattern suggests that a migratory emptying of the early eminential mantle possibly occurs, since there is no evidence that significant cell death occurs during development in this area (Trujillo 1982). The histogenetic pattern recalls that of the rhombic lip, a metamerically repeated hyperdorsal progenitor domain of multiple hindbrain rhombomeres, which also forms like the PThE next to roof plate chorioidal tissue. Various sizeable populations of precerebellar cells are produced along the rhombic lip, all of which massively migrate away tangentially into various hindbrain destinations, including the basilar pontine nuclei. The rhombic lip area itself finally appears largely depopulated.

Possible PThE neuronal migrations. Indeed, various earlier studies described possible neuronal migrations from the PThE into the subpallium (Hetzel 1974, 1975; Bulfone et al. 1995, 1999; Puelles et al. 2000; García-López et al. 2008; Abellán et al. 2010a; Medina and Abellán 2012). These studies identified as targets of eminential tangentially migrating cells the subpallial extended amygdala (EA) and the septocommissural area (Se). Recently we carried out an exhaustive analysis of the possible migrations that exit the avian PThE, using the PThE mantle marker *Tbr1*, and, following positive cells, described three distinct migratory streams: a subpial *peripeduncular stream*, an intermediate *juxtapeduncular stream* and a periventricular *eminentio-septal stream*. All three eminential streams exit in rostralward direction, as if the neurons were repelled by the caudally neighboring habenulo-thalamic microzone, or the intervening zona limitans. They penetrate both the peduncular and terminal alar hypothalamus (Puelles et al. 2012a; Puelles and Rubenstein 2015), where some elements become stabilized in the lateral hypothalamic area and in entopeduncular and supraoptic positions. Other migrated neuronal derivatives cross the hypothalamo-telencephalic border, targeting different territories in the preoptic and diagonal band areas, the extended amygdala and the commissural septum (Alonso et al. 2020).

Necessity of experimental corroboration. To test experimentally the results of that study, namely the conclusion that there exist three PThE-originated tangential migratory streams, we used the quail/chick chimeric experimental approach, in which orthotopic and isochronic grafts of the presumptive PThE territory of quail donors were placed into embryonic chick hosts. These fate-mapping experiments underpin the conclusion that a variety of *Tbr1-*expressing glutamatergic neurons observed in the hypothalamus, neighboring subpallium, and the septocommissural area come from the PThE. Migrated quail-tagged derivatives of these grafts were traced by specific anti-quail immunoreaction (QCPN antibody) at later stages. Some of these chimeric brains were also processed for *Tbr1* ISH, to compare the migration pattern of the graft-derived quail cells (detected by specific immunoreaction), with ectopic expression of the eminential *Tbr1* marker in the diverse target zones (obviously, the telencephalic pallium contains massive numbers of *Tbr1*-expressing cells, since this marker is also characteristic of the whole pallial mantle however, these telencephalic cells will lack the experimental quail tag).

## Materials and methods

### Animals

All experimental protocols and handling, use, and care of laboratory animals were conducted in compliance with the current normative standards of the European Union (Directive 2010/63/EU), the Spanish Government (Royal Decree 1201/2005 and 53/2013; Law 32/107), and with approval of the University of Murcia committee for animal experimental ethics.

Fertilized chick (*Gallus gallus*) and quail (*Coturnix coturnix japonica*) eggs from commercial sources were incubated at 37 °C in a forced-draft incubator with 65% humidity until the desired embryonic stage. Embryos were staged according to Hamburger and Hamilton (1951).

### Quail-chick chimeras

Orthotopic quail/chick grafts targeting the prethalamic eminence were performed at the fate-mapped diencephalo-telencephalic boundary neighborhood at stages HH10 or HH11. We used the chicken HH10 diencephalic fate maps of Trujillo and Alvarado-Mallart (1991) and García-López et al. (2004), as well as the HH10 secondary prosencephalon fate map of Pombero and Martinez (2009), and followed the grafting procedures of Couly and Le Douarin (1987) and Streit and Stern (2014). Since quail embryos develop somewhat faster than chick embryos, we synchronized donors and hosts by starting the incubation of quail eggs (donors) 2 or 3 h later than chick eggs (hosts).

Eggs were incubated in horizontal position to aid embryo location. Experiments were performed under sterile conditions. Several embryonic disks of quail donor embryos were extracted from the eggs, cutting them out with scissors. They were transferred to a Petri dish with the bottom covered with black paraffin wax and filled with Tyrode buffer supplemented with antibiotics. The quail embryonic discs were clamped to this base with small tungsten pins.

A chicken host was next prepared by opening a small window on the shell to access the embryo, and a small amount of Indian ink, diluted 1:10 in Tyrode buffer, was injected under the blastoderm with a glass micropipette, to facilitate visualization of the embryo. Finally, a portion of the vitelline membrane was removed over the prosencephalic vesicle with a sharpened tungsten needle. Tyrode solution was added as needed to evade drying out of the operation field. The desired portion of forebrain neuroepithelium was then carefully removed with the sharpened tungsten needle.

A grid with concentric circles and vertical/horizontal axes was inserted in one ocular of the operating microscope to normalize the dimension and location of grafts of the presumptive PThE and territories around it. The embryo was placed so that the vertical axis of the grid coincided with the embryonic midline, and the horizontal axis crossed the opto-diencephalic angle, a position that usefully subdivides the prosencephalon into four sectors (Fig. 1a). The radial distance between adjacent concentric circles in the grid measured 75 µm at 40X, the magnification used during microsurgery.

Immediately after a host chick embryo was ready, graft material from one donor embryo (corresponding to the form and site previously prepared in the host embryo) was excised with the tungsten needle, and it was transferred to the pre-prepared recipient chick embryo with a glass micropipette. The grafts were rectangular or wedge-shaped pieces of neuroepithelium, which were inserted carefully into the window previously opened in the host neural tube, maintaining its original rostro-caudal and dorso-ventral orientation. Finally, the window in the host eggshell was sealed with a piece of Parafilm secured with Scotch tape, and the host eggs were incubated further in horizontal position until sacrificed (normally at 10–11 days of incubation; see below).

### Tissue preparation

After the desired postoperative survival time, the chimeric embryos were extracted, and the heads were fixed in 4% paraformaldehyde fixative. Below 10 days of incubation the embryonic brains were dissected and fixed by immersion in 4% paraformaldehyde (diluted in 0.1 M phosphate-buffered, pH7.4; PB) at 4 °C for 48 h. Embryos at stage HH36 (10 days) or older up to hatchlings were deeply anesthetized with a euthanasic dose (240 mg/Kg of ketamine + xylazine), and then perfused transcardially with 0.75% NaCl saline solution, followed by phosphate-buffered 4% paraformaldehyde. Brains postfixed overnight in the same fixative solution were washed in 0.1 M, pH 7.4 phosphate-buffered solution, and processed for either paraffin, cryostat or vibratome sectioning. Brains to be cut as paraffin sections were dehydrated by immersion in successive ethanol dilutions of increasing percentage until 100% ethanol, and then cleared by immersion in xylene, after which they were transferred sequentially into low melting-point paraffin (1 h, 3 h, and overnight), and thereafter were embedded in paraffin blocks. Horizontal, sagittal or coronal 18 µm-thick sections were obtained in a microtome. Brains processed as vibratome free-floating sections were embedded in 4% low-melting point agarose (diluted in 0.1 M and pH 7.4 PBS). Horizontal, sagittal or coronal sections 80–90 µm-thick were obtained. Brains processed as cryostat sections were cryoprotected in 20% sucrose solution in PBS overnight at 4ºC and embedded in Tissue-Tek O.C.T. compound medium (Sakura). Blocks were frozen and stored at − 80 °C. Cryostat serial sections 20 µm-thick were cut in the transverse and horizontal planes, mounted in sets on Super Frost slides, and stored at − 80 °C until used.

### Immunohistochemistry

Sections were washed in PBS and then treated with 0.1% hydrogen peroxide in PBS for 1 h in the dark to inactivate endogenous peroxidase activity. After several rinses in PBT (PBS with 0.2% Triton X-100), sections were blocked with 0.5% goat serum, 0.2% bovine serum albumin (BSA) and 0.2% Triton X-100 (Sigma, St. Louis, MO, USA) in PBS for 4 h, and then, incubated overnight at 4 °C with monoclonal anti-quail QCPN antibody (Developmental Studies Hybridoma bank, Iowa City, IA, USA; Antibody Registry ID: AB_531886; dilution 1:5), prepared in the same blocking solution. This primary reaction was developed with biotinylated goat anti-mouse IgG secondary antibody (1:200, 2 h of incubation; Vector Laboratories, Burlingame, CA, USA), and then with streptavidin/horseradish peroxidase (HRP) complex (1:200, 2 h of incubation; Vectastain-ABC kit; Vector Laboratories, Burlingame, CA, USA). The histochemical detection of the peroxidase activity was carried out using 0.03% diaminobenzidine (DAB) and 0.005% H_2_O_2_. After immunoreactions, sections were mounted, dehydrated and then coverslipped with Eukitt (Fluka, Buchs, Switzerland).

### Antisera characterization

Quail-chick chimeric tissues were immunoreacted with the QCPN monoclonal antibody (Developmental Studies Hybridoma Bank, Iowa City, IA; dilution 1:5). This antibody was raised against quail wing bud ZPA (zone of polarizing activity) at stages HH21-24 and was shown to recognize species-specific quail cell antigens (Selleck and Bronner-Fraser 1995; Pombero and Martínez 2009). Pretreatment with BSA did not affect the immunostaining.

### In situ hybridization

Brains were processed for in situ hybridization with digoxigenin-UTP-labeled antisense riboprobes. Riboprobes for *Tbr1* (XM 003641638, positions 147-2032) were synthesized from plasmids kindly provided by J. L. Rubenstein (*Tbr1*). Hybridizations were done according to our standard protocol, as described by Ferrán et al. (2015a). As general in situ hybridization (ISH) controls, sense and antisense probes were applied to adjacent representative sections (the signal was present only with antisense probe), and some sections were processed without either sense or antisense probes, to check for possible background due to the other reactives used in the standard ISH procedure. To detect the hybridized product, sections were incubated overnight with alkaline phosphatase-conjugated antidigoxigenin Fab fragments (1:3.500, Roche Diagnostics, Manheim, Germany), and nitroblue tetrazolium/bromochloroindolyl phosphate (NBT/BCIP) was used as chromogenic substrate for the final alkaline phosphatase reaction (Boehringer, Mannheim, Germany).

### Imaging

Digital microphotographs were obtained with a Zeiss Axiocam camera (Carl Zeiss, Oberkochen, Germany) or with a ScanScope digital slide scanner (Aperio, Vista, CA, USA), and the images were corrected for contrast and brightness using Photoshop CS6 (Adobe Systems, San Jose, CA, USA). All plates were produced and labeled in Adobe Illustrator CS6 software (Adobe Systems, San Jose, CA, USA).

## Results

### Design of the prethalamic fate mapping experiments.

Here, we present experimental testing and tracing corroboration with the quail-chick chimeric grafting technique of our eminential migration hypothesis, which postulates that in chick embryos many eminential derivatives exit the original PThE microzone and invade rostrally placed hypothalamic and telencephalic targets. To this end, we designed isochronic and homotopic quail-chick chimeric experiments with a variety of unilateral grafts which encompassed all or a part of the presumptive PThE territory. The grafts always started at the dorsal midline of the neural tube (HH10-HH11) and extended more or less laterally in reference to a fate map schema for this stage derived from a synthesis of previously well-established fate-mapping results (Trujillo and Alvarado-Mallart 1991; García-López et al. 2004; Pombero and Martínez 2009) (Fig. 1a). The approximate extent over the fate map of our diverse grafts is shown in Fig. 1d at 40X magnification. The presumptive PThE field of quail and chick embryos is rather small and narrow, and has an oblique disposition relative to the midline (Fig. 1a, b). This obliquity, jointly with the expectable standard variation between different embryos, handicaps transplanting small PThE portions selectively enough that they are restricted to the prospective PThE. We circumvented this problem by mapping a diversity of larger forebrain sectors which theoretically overlapped each other (according to the fate map) over some part of the presumptive eminential territory, while including various other caudal and/or rostral neighboring non-PThE primordia, so that a reconstruction of the different affected areas could be made a posteriori*,* after histological analysis of the labelled areas (Fig. 1d). In most cases, the non-eminential parts of these grafts labelled parts of the brain that could be confidently identified as chorioidal, pallial, thalamic, habenular, or non-eminential prethalamic, whose histogenetic pattern is known and do not represent credible alternative sources for the migrated cells observed (Striedter et al. 1998; Cobos et al. 2001b; Ortino et al. 2003; Pombero and Martínez 2009; Garcia-Moreno et al. 2010). This experimental design was therefore able within limits to provide data which could be deduced to refer mainly given parts of the PThE primordium.

To assess the origin of quail cells found in the chimeric brains, we first checked any territories whose ventricular zone was itself graft-derived. The ventricular zone of the PThE, or a part of it, must derive from the graft in order to consider the possibility that ectopic quail cells may migrate from the eminence. As mentioned above, we also mapped according to their ventricular zone labelling any neural areas outside the PThE proper which were also graft-derived. In these cases, it was checked whether the expected pattern of non-eminential mantle labelling was present (normally, the adjacent mantle layer). In some cases, we produced chimeras affecting only the PThE neighbor areas as experimental controls. The resulting lack of labelled migrations thus excluded them as independent putative sources of migrated cells. Resulting chimeras were sacrificed in a number of steps between stages HH26 and HH40, to compare the gradual advance of quail-labelled migrated elements with that of *Tbr1*-expressing neurons (expected to coincide). In addition, we analyzed the moment in which each of the three migration streams begins, and the routes followed by the migrating cells. The extent of the grafts and the number of chimeric embryos processed are shown in Fig. 1 and Table 1, respectively.

**Table 1:** List of chimeric embryos selected for the present study. H, C, S: horizontal, coronal, sagittal section planes

Case	Stage at transplantation	Stage of fixation	Section type	Section plane	Histological treatment
*Q15 (cc)*	HH10	HH35	Paraffin	C	CV, QCPN
*Q16 (cc)*	HH10	HH35	Paraffin	H	CV, QCPN
*Q24*	HH10	HH35	Paraffin	C	CV, QCPN
*Q28 (cc)*	HH10	HH35	Paraffin	H	CV, QCPN
*Q29 (cc)*	HH10	HH35	Paraffin	C	CV, QCPN
*Q37*	HH10	HH35	Paraffin	H	CV, QCPN
*Q48*	HH10	HH35	Paraffin	S	CV, QCPN
*Q81 (cc)*	HH10	HH35	Paraffin	H	CV, QCPN
*Q96*	HH10	HH35	Paraffin	H	CV, QCPN
*Q131*	HH11	HH30	Paraffin	H	CV, QCPN
*Q151*	HH11	HH30	Paraffin	H	CV, QCPN
*Q153*	HH11	HH36	Paraffin	H	CV, QCPN
*Q192*	HH11	HH36	Paraffin	H	CV, QCPN
*Q196 (cc)*	HH11	HH36	Paraffin	H	CV, QCPN
*Q216 (cc)*	HH11	HH33	Paraffin	H	CV, QCPN
*Q224*	HH10	HH33	Paraffin	H	CV, QCPN
*Q226*	HH10	HH33	Paraffin	H	CV, QCPN
*Q236*	HH10	HH35	Paraffin	H	CV, QCPN
*Q241*	HH10	HH35	Paraffin	H	CV, QCPN
*Q258*	HH10	HH33	Paraffin	H	CV, QCPN
*Q261*	HH10	HH34	Paraffin	H	CV, QCPN
*Q269*	HH10	HH34	Paraffin	H	CV, QCPN
*Q278*	HH11	HH38	Vibratome	H	QCPN/*Tbr1*
*Q281* *Q292*	HH10 HH10	HH38 HH28	Vibratome Vibratome	S S	QCPN/*Tbr1* QCPN/*Tbr1*
*Q294*	HH10	HH35	Vibratome	S	QCPN/*Tbr1*
*Q301*	HH10	HH34	Vibratome	S	QCPN/*Tbr1*
*Q303*	HH10	HH34	Vibratome	C	QCPN/*Tbr1*
*Q304 (cc)*	HH10	HH30	Vibratome	S	QCPN/*Tbr1*
*Q320 (cc)*	HH11	HH37	Vibratome	H	QCPN/*Tbr1*
*Q323*	HH11	HH37	Vibratome	C	QCPN/*Tbr1*
*Q325*	HH11	HH39	Vibratome	S	QCPN/*Tbr1*
*Q327 (cc)*	HH10	HH35	Vibratome	H	QCPN/*Tbr1*
*M6*	HH10	HH26	Vibratome	C	QCPN/*Tbr1*
*M15*	HH10	HH25	Vibratome	S	QCPN/*Tbr1*
*M25*	HH10	HH38	Cryostat	C	QCPN/*Tbr1*
*M33*	HH10	HH36	Cryostat	C	QCPN/*Tbr1*
*M45*	HH11	HH39	Vibratome	C	QCPN/*Tbr1*
*M51 (cc)*	HH10	HH35	Vibratome	C	QCPN/*Tbr1*
*M69*	HH10	HH34	Cryostat	H	QCPN/*Tbr1*
*M88*	HH10	HH30	Cryostat	C	QCPN/*Tbr1*
*M106*	HH10	HH40	Cryostat	C	QCPN/*Tbr1*
*M139*	HH10	HH40	Cryostat	C	QCPN/*Tbr1*

*CV* cresyl-violet stain, *cc* control chimera

### Analysis of chimera brains

#### Short-survival chimeras: fixation at stages HH25/26 to HH30

**Fig. 2:**
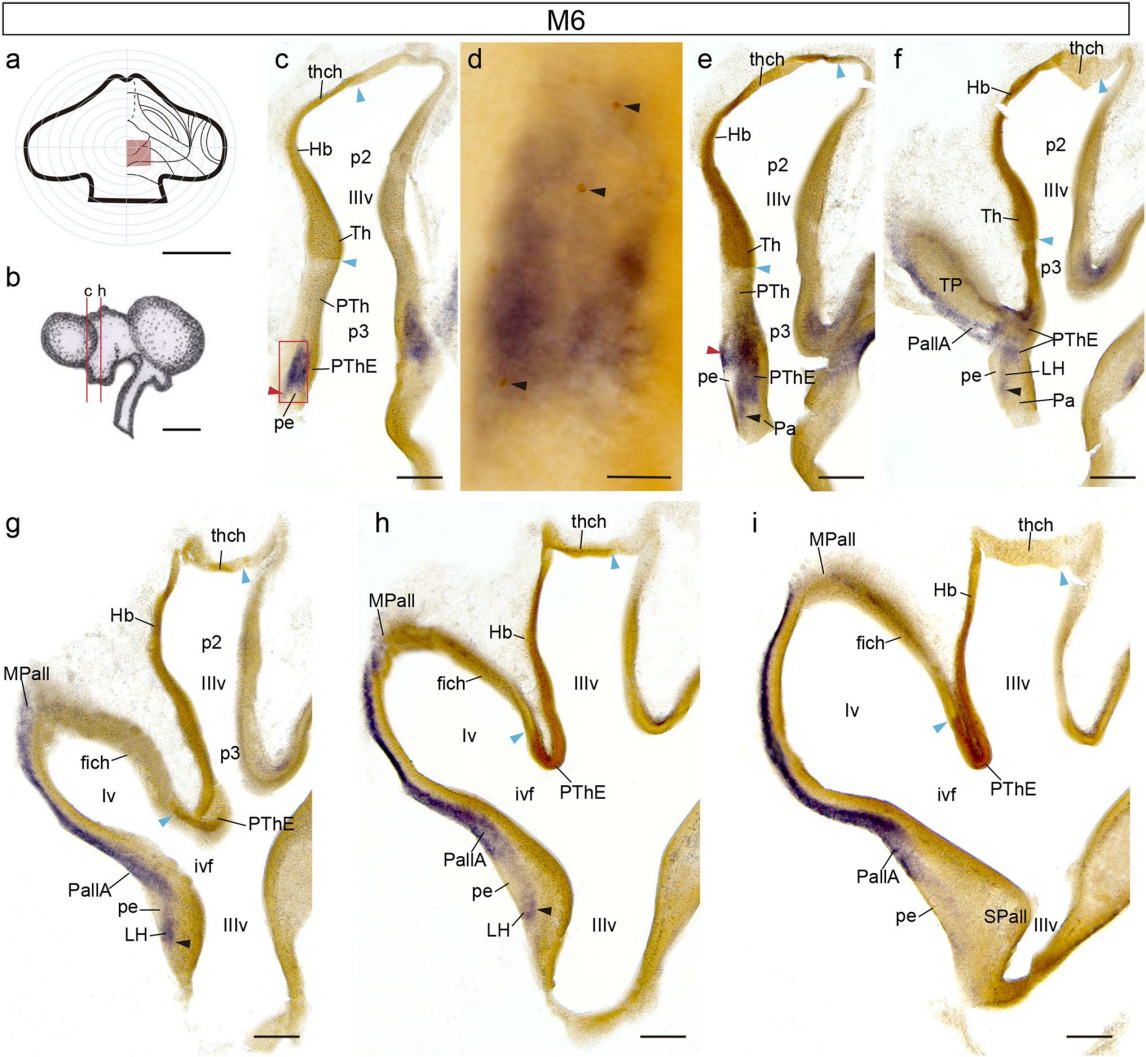
Coronally sectioned chimeric embryo (case M6), fixed at stage HH25/26, and processed for *Tbr1* ISH and QCPN IMR. **a** Drawing of graft extent relative to the prosencephalic fate map. **b** Schema of the M6 chimeric brain, illustrating the plane of the sections shown in (**c**, **e–i**). **c, e–i** Caudorostral series of coronal sections through the di-telencephalic transition. Blue arrowheads indicate the borders of the graft at the ventricle, red arrowheads point to the peripeduncular stream, and black arrowheads point to the juxtapeduncular stream. **d** Magnification of the area framed in (**c**). Black arrowheads point to quail-derived cells in the PThE. Scale bars in **a** 250 μm, **b**, 2.5 mm, **c, e-i**, 150 μm, and **d**, 50 μm

Our first example of short-survival chimeras is case M6, fixed at stage HH25/26 (4.5–5 d.i.o.), cut coronally (levels of Fig. 2c–h schematically indicated in Fig. 2b), and processed for *Tbr1* ISH and QCPN immunoreaction (IMR). The estimated graft extent relative to the fate map appears in Fig. 2a. At HH25/26, the grafted neuroepithelium area appears as a dense brown domain (enclosed between two blue arrowheads; Fig. 2c–h). Quail-labelled graft derivatives include the di-telencephalic chorioidal tela (Fig. 2c–f), the dorsal part of the PThE (Fig. 2f–h; note partially unlabelled ventricular zone of PThE in Fig. 2c–e), and parts of the habenula and thalamus (Hb, Th; Fig. 2c–h). *Tbr1* ISH signal appears throughout the eminential mantle layer, as well as at a subpial aggregation of *Tbr1*-positive cells on top of the cerebral peduncle, suggestive of an incipient *peripeduncular migratory stream* (red arrowheads; Fig. 2c, c’, d; the peduncle [pe] appears as a superficial packet of unstained fibers). Sections through the peduncular hypothalamus show a continuity of the *Tbr1*-positive PThE mantle deep to the peduncle with blue cells within the lateral hypothalamus, indicating presence of an advanced *juxtapeduncular migratory stream* at this stage, which occupies the lateral hypothalamus mantle superficial to the paraventricular hypothalamic nucleus (black arrowheads; Fig. 2e–g). However, QCPN-labelling shows no quail migrated cells at the lateral hypothalamus, and only a few labelled cells were observed at the peripeduncular stream (small black arrows in Fig. 2d). It may be deduced from this discrepancy at the lateral hypothalamus that the earliest cells that exit from the PThE largely must originate from its *unlabelled* ventral part. The dorsal PThE part labelled selectively in this experiment contributes few if any cells to juxtapeduncular (hypothalamic) migrated PThE cells at this stage but enters incipiently the peripeduncular stream. This result suggests that the juxtapeduncular stream slightly precedes the peripeduncular stream at this stage.

**Fig. 3:**
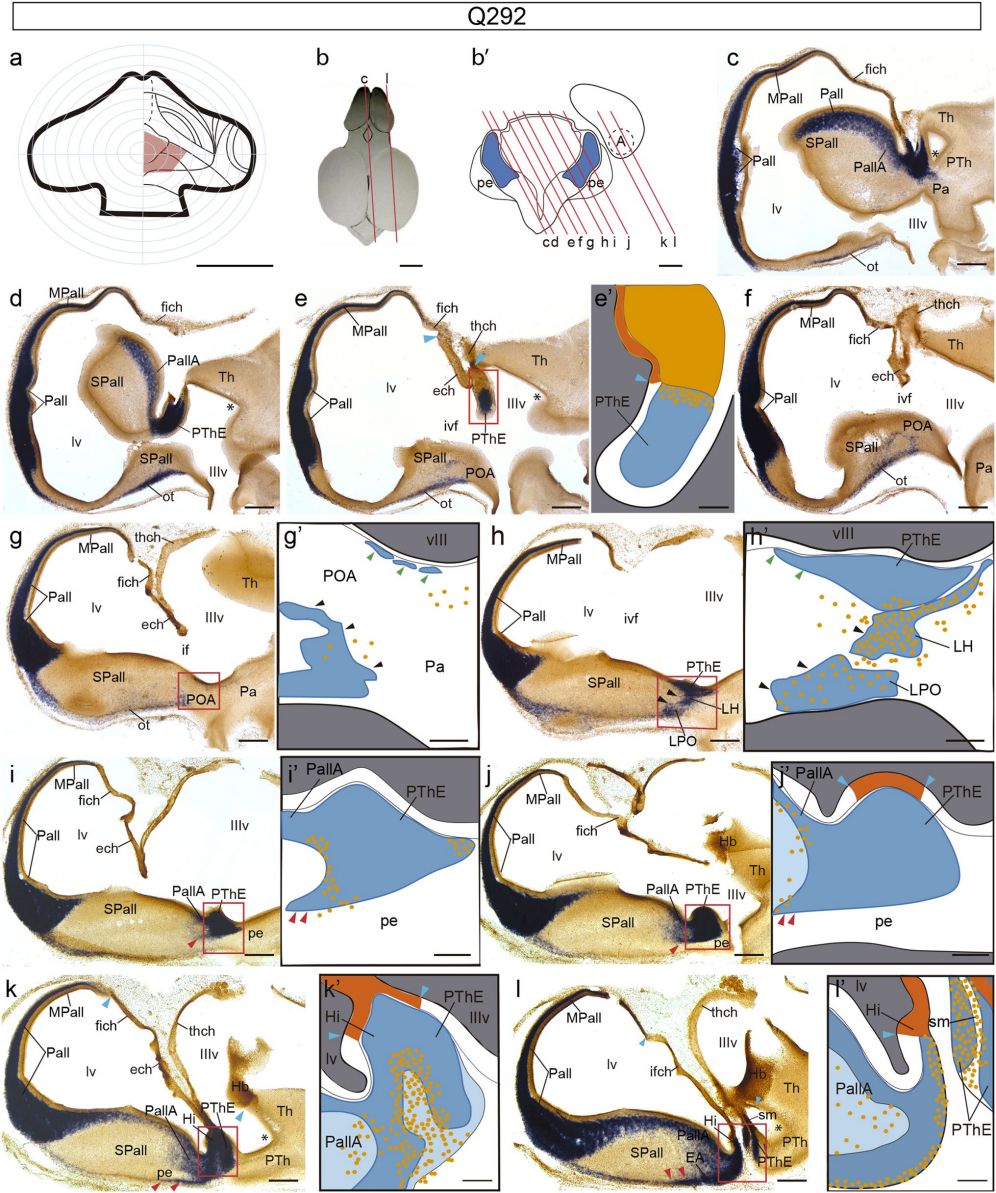
Sagittal-obliquely sectioned chimeric embryo (case Q292), fixed at stage HH28, and processed for *Tbr1* ISH and QCPN IMR. **a** Drawing of graft extent relative to the prosencephalic fate map. **b–b’** Schemata of the Q292 chimeric brain, illustrating the oblique plane of these sections. **c–l** Medio-lateral series of oblique sections through the prosencephalon. **e’**, **g’–l’** Schematic drawings of the areas framed in red in (**e**, **g**–**l**), respectively. Blue arrowheads indicate the ventricular borders of the graft. Red arrowheads point to the peripeduncular stream. Black arrowheads point to the juxtapeduncular stream. Green arrowheads point to the incipient eminentio-septal stream. Scale bars in **a** 250 μm, **b**, 2.5 mm, **b’**, 250 μm, **c–j**, 150 μm and **e’**, **g’–l’**, 50 μm

**Fig. 4:**
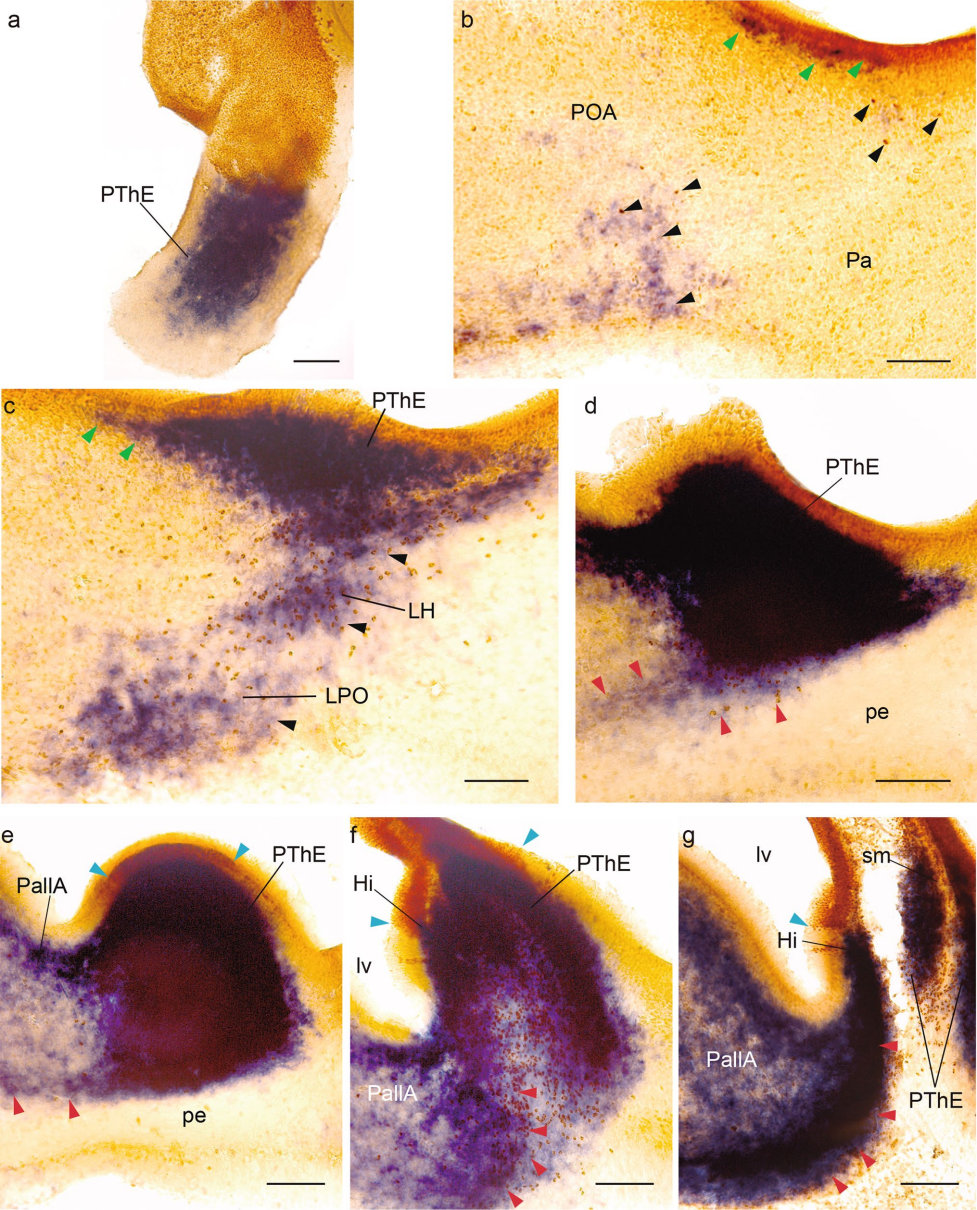
Higher magnification images of the areas framed in the sections in Fig. 3**e, g–l**. Blue arrowheads indicate the ventricular borders of the graft. Red arrowheads point to the peripeduncular stream. Black arrowheads point to the juxtapeduncular stream. Green arrowheads point to the eminentio-septal stream. Scale bars in **a-g**, 50 μm

A second example of our short survival eminential chimeras is case Q292, sacrificed at stage HH28 (5.5–6 d.i.o.; graft map in Fig. 3a; section orientation in Fig. 3b, b’; grafted derivatives in Fig. 3c–l, with schematic maps of grafted cells in Fig. 3c’–l’, and high magnification microphotographic details in Fig. 4a–g). This brain, doubly reacted for *Tbr1* ISH and QCPN IMR, was accidentally sectioned in an oblique sagittal plane (see Fig. 3b, b’). We were guided in our interpretation of these sections by the known *Tbr1* pattern (Alonso et al. 2020). The series shown starts close to the midline at the control side (Fig. 3c), where we see continuity of eminential *Tbr1* signal with that of the telencephalic caudal ‘ventricular ridge’ pallium (including periventricular parts of pallial amygdala; PallA); separate cortical pallial parts (Pall/MPall) are also labelled with *Tbr1*, as well as subpial migrated pallial cells in the subpallial olfactory tuberculum (SPall; ot). The diminishing contralateral PThE is seen again in Fig. 3d, e, after which we have two other sections illustrating the (QCPN-labelled) median chorioidal roofplate (ech; Fig. 3f, g; compare Fig. 3b’). The experimental PThE is then sectioned first at its ventral end (Fig. 3h; compare Fig. 3b’), and the eminential section series then proceeds dorsalward across PThE, showing its relationships with the cerebral peduncle and associated labelled migration streams (PThE, pe; black and red arrowheads; Fig. 3h–l). At levels through Fig. 3k, l, the experimental PThE reaches its dorsal contact with the chorioidal roof (ech; Fig. 3l; compare Fig. 3b’). The non-eminential prethalamus appears laterally to the peduncle (PTh; Fig. 3k, l). Due to their obliquity, these last sections also provide an unusual insight on the neighborhood relationships of PThE with the pallial amygdala, as well as with the neighboring caudolateral end of the hippocampus, at the caudal tip of the hemisphere (PallA, Hi; Fig. 3k, l).

We mapped schematically the location of labelled quail cell nuclei at ventricular and mantle zone levels (Fig. 3e’–l’) and we show in Fig. 4 correlative microphotographic details of framed areas (Fig. 4a–g; note sometimes the QCPN-labelled brown cell nuclei are partly hidden by the blue *Tbr1* ISH signal where it is massive, as occurs inside the PThE mantle; nevertheless, the brown label usually can be detected at high magnification). As regards the grafted domain, a large part of the diencephalic (eminential, ech; thalamic, thch) and telencephalic/fimbrial (fich) chorioidal tela of the transplanted hemisphere was graft-derived (ech, thch, fich; Fig. 3e–g, i–l), as well as most of the right habenula (Hb; Fig. 3j–l), and the whole dorsal part of the PThE (compare Fig. 3a; QCPN labelling of the ventricular zone is seen at levels of Figs. 3j, j’, k, k’ and 4e, f). Ventral section levels through the PThE did not show clearcut ventricular zone labelling (Fig. 3h, h’, i, i’), though migrated labelled cells were observed there in the mantle. The non-eminential prethalamus was not included in the graft (PTh; Fig.3a; k, l). There was also no QCPN-labelled ventricular zone at the pallial amygdala or hippocampus, or any other part of the telencephalic pallium.

According to these data, the Q292 graft included massively the dorsal part of the PThE, in contrast to the partial dorsal labelling observed in the M6 case, which also had a shorter survival. By stage HH28 many *Tbr1*- and QCPN-positive eminential quail cells have moved outside the PThE, following either the juxta- or peripeduncular streams described previously (Alonso et al. 2020). The double-labelled juxtapeduncular stream is best developed and is found typically just medial (deep) to the cerebral peduncle, the site of the lateral hypothalamus; some of its cells reach the lateral preoptic area (black arrowheads; POA, LPO, LH; Fig. 3g, h). The *peripeduncular stream* is less advanced; most of the corresponding double-labelled cells appear aggregated between the PThE mantle and the back of the peduncle, where this stream begins (pe; red arrowheads in Fig. 3i, j). However, more lateral sections tangent to the outside of the peduncle show labelled cells of this stream squeezing subpially around the peduncle and advancing into the basal telencephalon. Some labelled cells even seem to have reached the extended amygdala (red arrowheads, pe, EA; Fig. 3k, l). However, more medial sections in Fig. 3i/i’, j/j’ and correlative details in Fig. 4d, e show that practically no *Tbr1*- or QCPN-labelled cells can be seen subpially ventrally to the peduncle, indicating that migrating peripeduncular eminential cells do not yet reach the preoptic and diagonal areas at stage HH28, as they will do at later stages (Alonso et al. 2020). There is practically no indication of the third, eminentio-septal migratory stream at this stage, with the possible exception of a few double-labelled cells observed outside of the PThE, next to the paraventricular hypothalamic ventricular zone (Figs. 3g, g’ and 4b). These may represent the earliest sign of the eminentio-septal stream.

#### Intermediate survival chimeras: fixation at stages HH33 to HH38

**Fig. 5:**
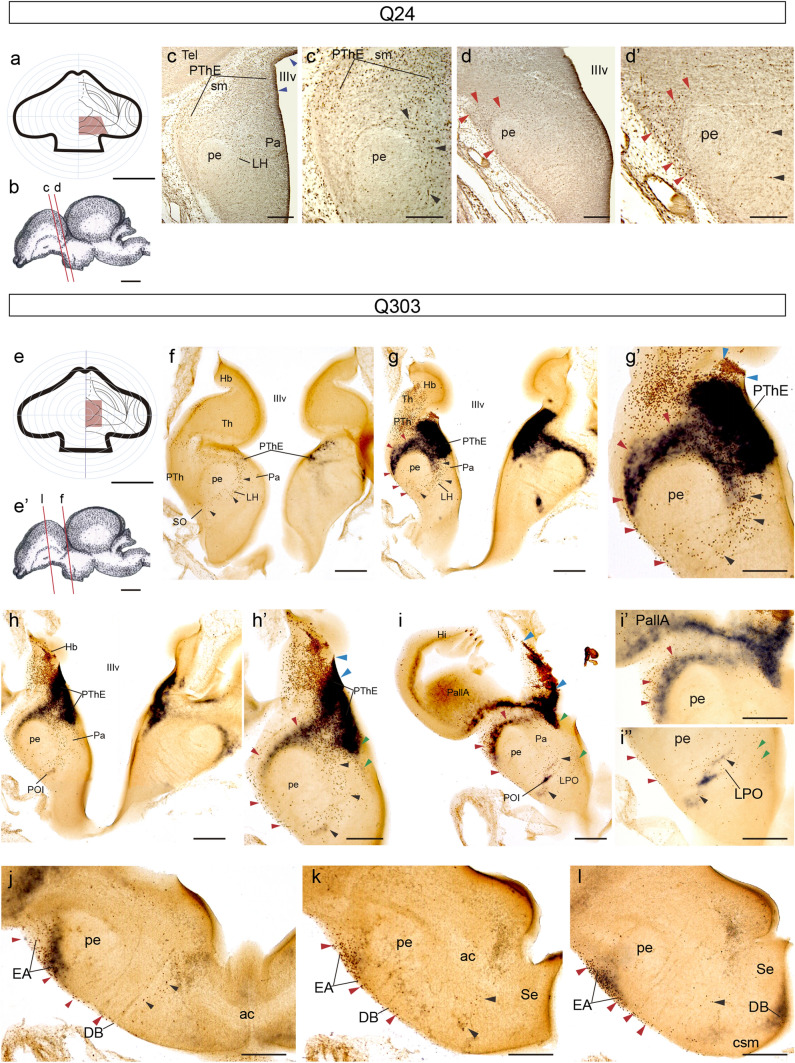
Images of cases Q24 and Q303. Both chimeric brains were sectioned in a plane orthogonal to the peduncle. Q24 was only processed for QCPN IMR, and Q303 was processed for *Tbr1* ISH and QCPN IMR. **a** Drawing of Q24 graft extent relative to the prosencephalic fate map. **b** Schema of the Q24 chimeric brain, illustrating the plane of the sections shown in (**c**–**d**). **c** The section is perpendicular to the peduncle (pe), thus coinciding roughly with the direction of the eminential migration streams; alar hypothalamic level. **c’** Higher magnification of **c** showing the quail-labelled juxtapeduncular stream (black arrowheads). **d** More dorsal section passing through the di-telencephalic transition. **d’** Higher magnification of **d** showing the quail-labelled elements in the juxtapeduncular and peripeduncular streams (black and red arrowheads, respectively). **e** Drawing of Q303 graft extent relative to the prosencephalic fate map. **e’** Schema of the Q303 chimeric brain, illustrating the plane of the sections shown in (**f**–**l**). **f–i** Ventrodorsal series through the PThE showing its neighborhood relationships with the PTh, Pa and PallA. **g–i’’** Higher magnification of **g–i** showing details of the peripeduncular, juxtapeduncular and eminentio-septal streams. **j–l** Three consecutive sections through the subpallium at the level of the commissural septum. Blue arrowheads indicate the ventricular borders of the graft. Red arrowheads point to the peripeduncular stream. Black arrowheads point to the juxtapeduncular stream. Green arrowheads point to the eminentio-septal stream. Scale bars in **a** and **e**, 250 μm, **b** and **e’**, 2.5 mm, **c** and **d**, 150 μm, **c’** and **d’**, 50 μm, **f–i**, 150 μm, **g’–i’’**, 50 μm, **j–l**, 150 μm

The coronally sectioned chimeric brains Q24 and Q303 (fixed at stage HH33/34-8 d.i.o.), illustrate the behaviour of migrating PThE cells at these stages, particularly with regard to their relationship with the peduncle. The HH33 Q24 chimera (map in Fig. 5a; ventricular zone marked between blue arrowheads in Fig. 5c; section plane for 5c, d in Fig. 5b) was one of our earliest experiments, performed before we had the possibility to perform in situ hybridization in our laboratory, and was therefore only labelled with QCPN IMR. This labelling revealed as grafted quail derivatives a small dorsocaudal portion of the PThE (Fig. 5a, c), jointly with a part of the adjacent di-telencephalic chorioidal tela (not shown), and parts of the rostral habenula (Hb) and thalamus (Th) (Fig. 5a). Quail-derived eminential cells fill up the dorsal deep and superficial mantle of the PThE (strata separated by the stria medullaris tract; PThE, sm; Fig. 5c, c’). At the caudal level shown in Fig. 5c, either the mass of peripeduncular cells or a part of non-eminential prethalamus is visible superficially to the peduncle, but this locus contains practically no quail-derived cells (detail in Fig. 5c’). At this level, numerous QCPN-labelled quail cells found within the lateral hypothalamus characterize the juxtapeduncular migratory stream, settling down in the lateral hypothalamus, medially to the peduncle (black arrowheads, LH, pe; Fig.5c, c’). A more rostral section level nevertheless shows abundant quail-derived cells in peripeduncular position; these are also found partly superficially to the peduncle; some juxtapeduncular migrated cells are also seen nearby (red and black arrowheads; pe; Fig.5d, d’).

The likewise coronally sectioned Q303 chimera (fixed at stage HH34 -8 d.i.o.) was processed for *Tbr1* ISH and QCPN IMR (operation map in Fig. 5e; section levels in 5e’; quail ventricular zone between blue arrowheads in Fig. 5g, g’–i). In this chimeric brain the di-telencephalic chorioidal tela, a dorsal part of thalamus (Th) and habenula (Hb), a small dorsal part of the PThE, and a dorsalmedial portion of the medial pallium (Hi, MPall) derived from the quail graft (Fig. 5e; blue arrowheads in Fig.3g’, h’, i’). Numerous quail cells appear dispersed within the eminential *Tbr1*-positive mantle, apparently migrated from the small quail ventricular fragment observed dorsocaudally (Fig. 5g, g’, h, h’). Likewise, a number of quail cells have left by this stage the labelled eminential territory, following the three migratory routes described (juxtapeduncular, peripeduncular, and eminentio-septal streams; Alonso et al. 2020). As regards the peripeduncular stream quail *Tbr1*-positive cells clearly surround the peduncle, reaching rostrally the extended amygdala (EA), which already appears populated by numerous quail cells (red arrowheads; pe, EA; Fig. 5g, g’, h’, i, i’, j–l). In addition, some quail cells detach from the peripeduncular migratory stream and follow a subpial route into the Dg area (red arrowheads; DB; Fig. 5j–l). The juxtapeduncular migratory stream is also populated by numerous quail eminential cells which have followed a caudo-rostral pathway reaching the LH, and the LPO (Fig. 5g, g’, h’, i, i’, i’’, j–l). Some of these quail cells converge as well towards the anterior commissure (ac; Fig. 5k). The eminentio-septal migratory stream is not yet massive at this stage, but we observed some periventricular cells heading periventricularly rostralwards from the PThE, approaching the area of the anterior commissure, through which they will enter the septocommissural region (green arrowheads; ac; Fig.5h’, i, i’, i’’, j–l). *Tbr1* expression mixed with QCPN-positive cell nuclei jointly corroborates the rostral and lateral contact of the PThE with the caudal hippocampus (Hi) and the pallial amygdala (PallA) (Fig.5i, i’).

**Fig. 6:**
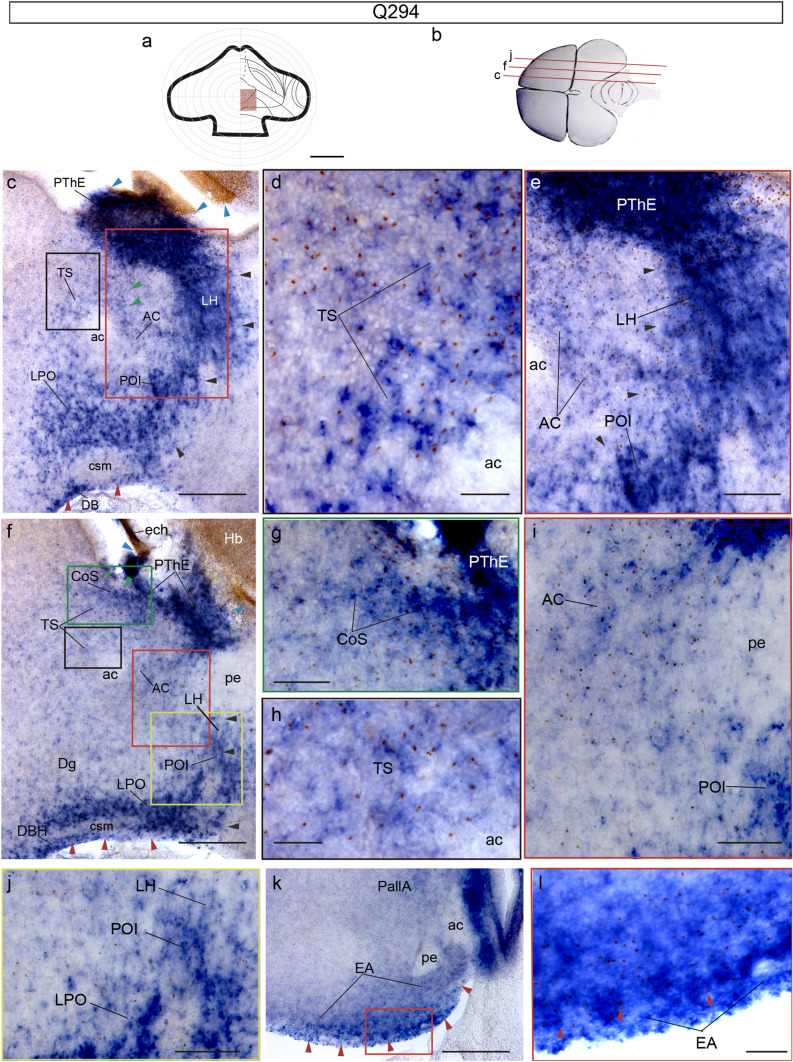
Case Q294, sacrificed at HH35, processed for *Tbr1* ISH and QCPN IMR, and sectioned sagittally. **a** Drawing of the graft extent relative to the prosencephalic fate map. **b** Schema of the Q294 chimeric brain, illustrating the plane of the sections shown in (**c**–**l**). **c** Detail of the PThE and the juxtapeduncular and eminentio-septal streams at the commissural septum level. **d-e** Higher magnification of the areas framed in a black and a red square in (**c**); observe quail cells (red dots) in the *Tbr1-*positive area. **f** Detail of a parasagittal section alevel with the peduncle entering the subpallium. **g–j** Higher magnifications of colored framed areas in (**f**). **k** Section of the telencephalon at the level of the extended amygdala (EA). **l** Higher magnification of the red framed area in (**k**), where red arrowheads point to peripeduncular migrated quail cells. Blue arrowheads indicate the ventricular borders of the graft. Red arrowheads point to the peripeduncular stream. Black arrowheads point to the juxtapeduncular stream. Green arrowheads point to the eminentio-septal stream. Scale bars in **a**, 250 μm, **b**, 2.5 mm, **c**, **f** and **k**, 150 μm, **d**–**e**, **g–j and l**, 50 μm

We will examine next the Q294 chimera, which was sacrificed at HH35 (8.5–9 d.i.o.), sectioned sagittally, and doubly processed for *Tbr1* ISH and QCPN IMR. This case consists of a very small eminential graft which also affected the chorioidal roof, the Hb and Th (Fig. 6a). The graft derivatives include a dorsocaudal portion of the PThE, a rostromedial part of Hb and Th, as well as part of the diencephalic chorioidal tela (Fig. 6a–l). The two medial sagittal sections shown (Fig. 6c,f and color-coded framed details 6d, e/g–j) illustrate double-labelled migrated cells (marked by brown nuclei and blue *Tbr1*-expressing cell bodies) along the three eminential streams. The juxtapeduncular migratory stream (black arrowheads) expands massively the PThE ventralwards and occupies the local lateral hypothalamus at hypothalamic paraventricular area levels (LH; Pa; Fig. 6c, e, f, j); some of its pioneering cells reach the superficial lateral preoptic area, wherein the more compact ‘preoptic island’ described in Alonso et al. (2020) is already distinguished (LPO, POI; Fig. 6e, f, i, j). The eminentio-septal migratory stream (green arrowheads; Fig. 6f) appears at this stage as a relatively compact band of deep periventricular double-labelled cells sorting out rostralward from the PThE; it enters the septocommissural domain represented by the anterior commissure (ac), its associated anterior commissure nucleus (AC), and the septal triangular nucleus (TS). The more compact dorsal elements of this stream are interpreted as the primordium of the commissural septal nucleus (CoS; Fig. 6f, g). The peripeduncular migratory stream is shown here only after it finishes its peripeduncular course proper (i.e., after coursing subpially all around the peduncle), and then passes subpially (superficially) from the lateral preoptic area into the diagonal band (red arrowheads, LPO, DB; Fig. 6c, f), on its way to the abundantly double-labelled extended amygdala (EA; the latter is shown in the more lateral sagittal section illustrated in Fig. 6k, l).

#### Long survival chimeras: fixation at stages HH38 to HH40.

**Fig. 7:**
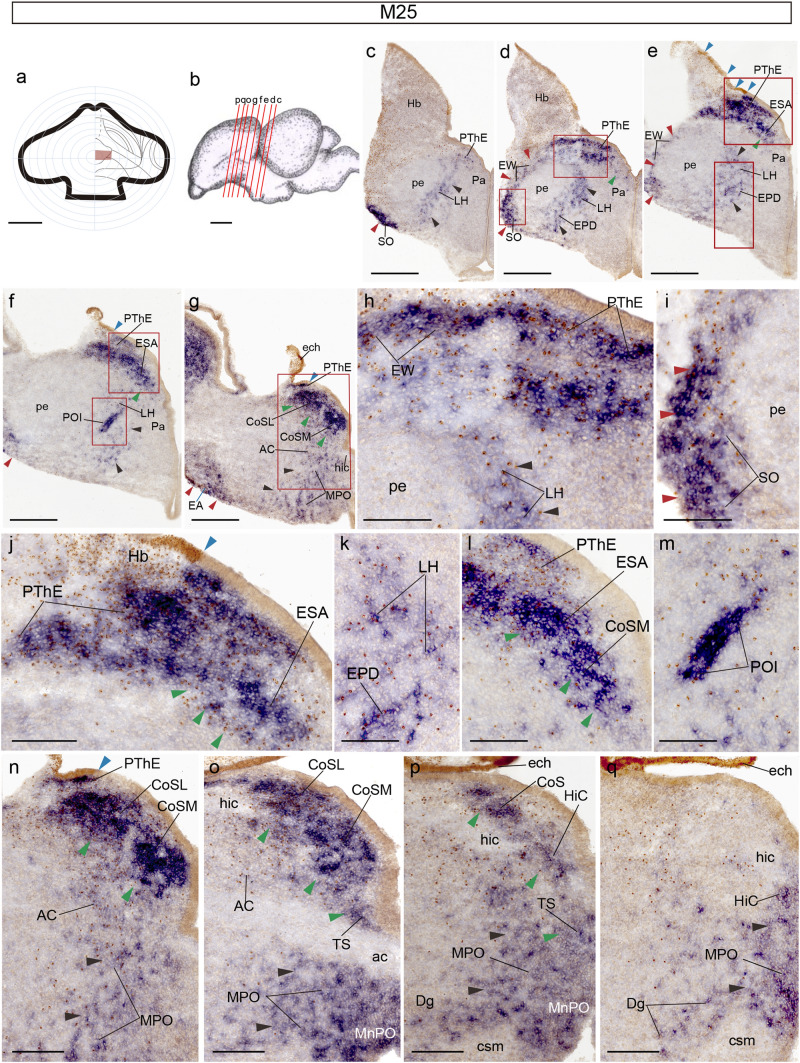
Case M25, sacrificed at HH38, processed for *Tbr1* ISH and QCPN IMR, and sectioned in a plane orthogonal to the peduncle. **a** Drawing of graft extent relative to the prosencephalic fate map. **b** Schema of the M25 chimeric brain, illustrating the plane of the sections shown in (**c**–**q**). **c–g** Ventrodorsal series through the diencephalic-telencephalic transition. **h–i** Higher magnifications of red framed areas in (**d**). **j–k** Higher magnification of red framed areas in (**e**). **l–m** Higher magnification of red framed areas in (**f**). **n** Higher magnification of red framed area in (**g**). **o–q** Details of three consecutive sections situated immediately dorsal to (**n**). Blue arrowheads indicate the ventricular borders of the graft. Red arrowheads point to the peripeduncular stream. Black arrowheads point to the juxtapeduncular stream. Green arrowheads point to the eminentio-septal stream. Scale bars in **a**, 250 μm, **b**, 5 mm, **c-g**, 150 μm, **h-m**, 50 μm, **n-q**, 150 μm

Long survival experiments display advanced stages of the studied tangential migrations and allow more precise anatomic identification of the labelled nuclei. Chimera M25 (Fig. 7a) was sacrificed at HH38 (12 d.o.i.), sectioned roughly orthogonal to the peduncle (Fig. 7b) and double-labelled with Tbr1 ISH and QCPN IMR (Fig.7c–q). The graft includes the local chorioidal tela (Fig. 7g, o–q), as well as a rostrodorsal portion of Hb (Fig. 7e, j), and the dorsalmost part of the presumptive PThE territory (Fig. 7a, g, n). It is similar in extent to the Q294 graft. Interestingly, ventral parts of the PThE whose ventricular zone is QCPN-negative (host-derived) show abundant intermixed quail cells in the mantle, suggesting that at least some migrating cells course through the PThE itself before entering one of the external migratory streams (Fig. 7h, j). Derivatives of the eminential juxtapeduncular and peripeduncular streams start to become histologically stabilized and their previous connective bridges with the PThE start to disappear (red and black arrowheads; Fig. 7c–g). In contrast, the more retarded eminentio-septal still maintains a straightforward migratory appearance well connected to the PThE (green arrowheads; Fig. 7e–g). The juxtapeduncular stream populates the LH, LPO, POI, MPO and MnPO target areas (Fig. 7c–h, k, m, n–q). In more rostral sections, scattered cells also appear in the Dg area (Dg; Fig. 7p, q). As regards peripeduncular stream derivatives, few double-labelled cells remain dorsolateral to the peduncle, forming what we called ‘eminential wings’ (red arrowheads, EW; Fig. 7d, e; Alonso et al. 2020), whereas denser labelled elements of the extratelencephalic portion of this stream are seen ventrolaterally to the peduncle, at the site tentatively identified as supraoptic nucleus (red arrowheads, SO; Fig .7c, d, i). The telencephalic portion shows labelled derivatives at the extended amygdala (red arrowheads, EA; Fig. 7g) and may contribute also to the preoptic and diagonal areas (not shown). The nuclear derivatives of the eminentio-septal stream, forming the eminentio-septal area (ESA) rostral to the PThE, are still immature at this stage on their way to the septocommissural area (green arrowheads, ESA; Fig. 7e, f, j, l). Many double-labelled cells start to aggregate at the primordia of the lateral and medial CoS nuclei (green arrowheads, CoSL, CoSM; Fig. 7g, n–p). In contrast, the nucleus of the hippocampal commissure and the triangular septal nucleus only show a few dispersed cells (green arrowheads, HiC, TS; Fig. 7o–q).

**Fig. 8:**
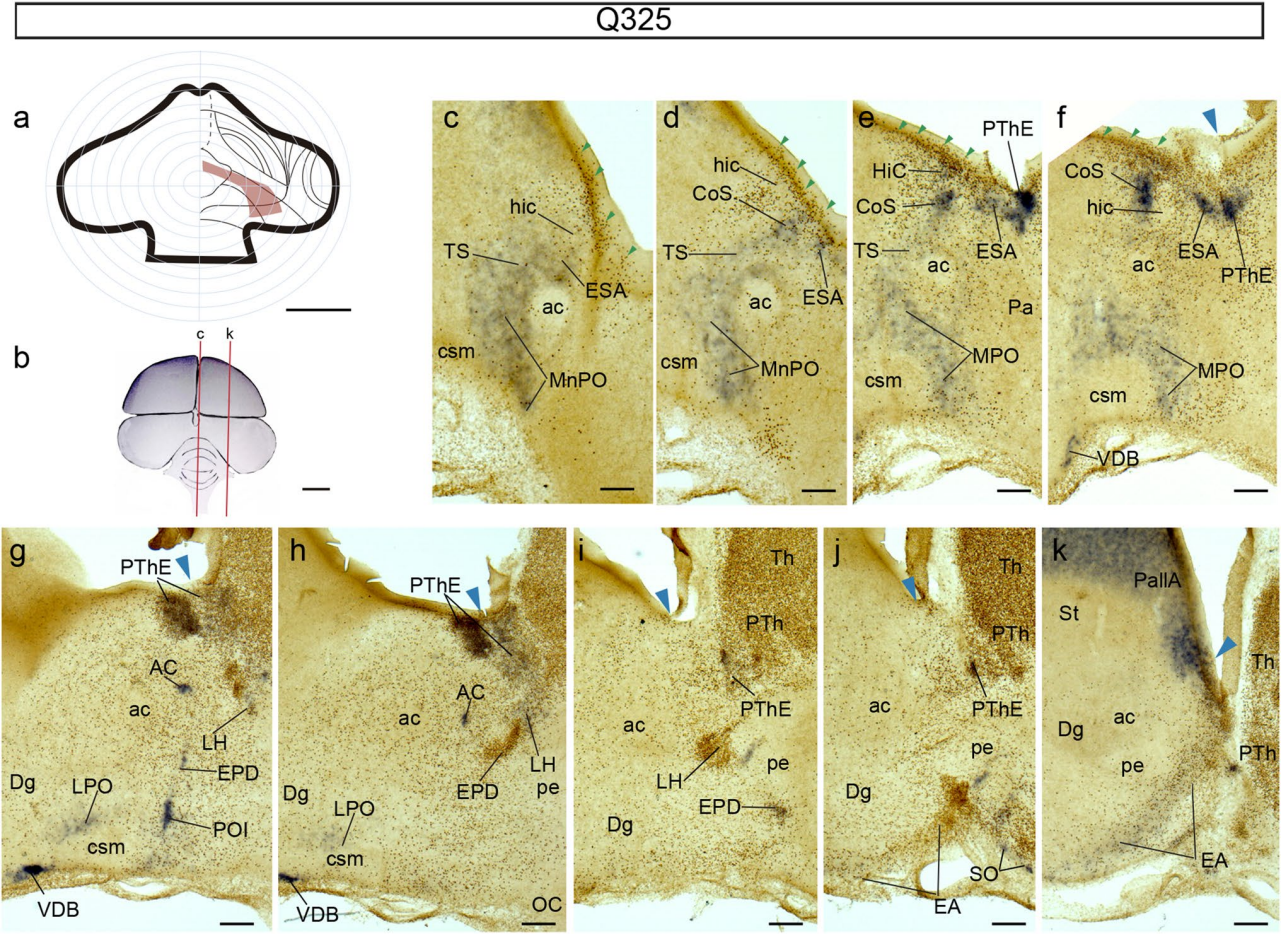
Case Q325, sacrificed at HH39, processed for *Tbr1* ISH and QCPN IMR, and sectioned sagittally. **a** Drawing of graft extent relative to the prosencephalic fate map. **b** Schema of the Q325 chimeric brain, illustrating the plane of the sections shown in (**c**–**k**). **c–k** Medio-lateral series of sections roughly at the diencephalo-telencephalic transition. Blue arrowheads indicate the ventricular borders of the graft. Green arrowheads point to the eminentio-septal stream. Scale bars in **a**, 250 μm, **b**, 5 mm, **c–k**, 150 μm

To check whether the septocommissural TS and HiC nuclei, the preoptic MnPO and MPO nuclei, and the diagonal band nuclei receive more massively migrated cells when the topologically dorsal and ventral regions of the PThE are jointly grafted, we performed some grafts covering the whole dorsoventral extent of the presumptive eminential territory. These chimeras usually also included small parts of the caudal MPall (Hi) and/or the presumptive PallA, or of the non-eminential central alar prethalamic territory. A representative example of such experiments is case Q325, sacrificed at HH39 (13 d.o.i.; Fig. 8a, b). At this stage, eminential migrations are very advanced, or have ended (Alonso et al. 2020). In the Q325 chimera almost all the presumptive territory of the PThE was grafted, only excepting its caudalmost portion next to the Hb (Fig. 8a). Parts of alar prethalamus, thalamus, and PallA, were labelled as well (Fig. 8a). In this chimeric brain there is a striking rostral dispersion of PThE-originated quail cells in the septocommissural periventricular stratum (small green arrowheads in Fig. 8c–f; the grafted PThE appears in Fig. 8g, h). We interpret this pattern as quail cells migrating through the deep eminentio-septal stream under the interventricular foramen. All nuclei previously described as recipients of eminential migrating cells (Alonso et al. 2020) display *Tbr1*-labelled quail cells (Fig. 8c–k). However, the MnPO and MPO nuclei are distinct in not showing massive populations of graft-derived quail cells, in comparison to their level of *Tbr1* expression. The graft left out a caudal eminential region from which these prospective preoptic cells may have originated (Fig. 8a); however, we cannot rule out the possibility that these preoptic nuclei, similarly as the HiC, Dg and DB nuclei, may receive migrated *Tbr1*-positive cells from the telencephalic pallial region (Pombero et al. 2011; Alonso et al. 2020). In this chimera the subpallium appears invaded by numerous dispersed quail cells (Fig. 8h–m). These cells do not express *Tbr1*, a result which suggests an oligodendrocyte or astroglial nature of these dispersed elements (see Cobos et al. 2001a, b; Olivier et al. 2001).

#### Analysis of control experiments

**Fig. 9:**
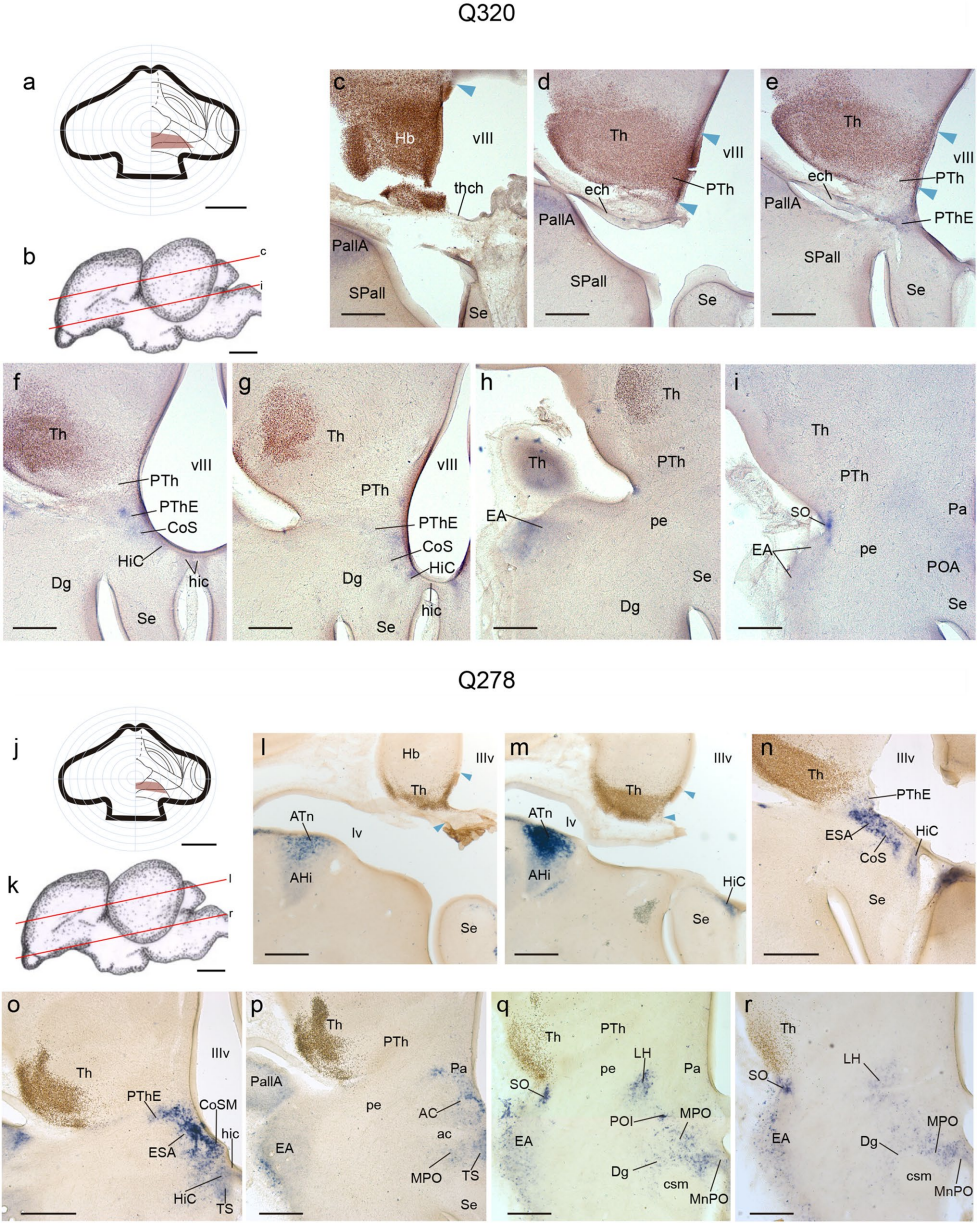
Control cases Q320 (**a–i**) and Q278 (**j–r**), sacrificed at HH37 and HH38, respectively, processed for *Tbr1* ISH and QCPN IMR, and sectioned in a standard horizontal plane. **a** Drawing of the Q320 graft extent relative to the prosencephalic fate map, inciding on prospective habenula, thalamus and a small part of non-eminential prethalamus. **b** Schema of the Q320 chimeric brain, illustrating the plane of the sections shown in (**c–i**). **c–i** Dorso-ventral section series across the eminential region and some targets of eminential migrations of Q320, showing lack of QCPN-labeled cells. **j** Drawing of the Q278 graft extent relative to the prosencephalic fate map, inciding on prospective habenula and thalamus. **k** Schema of the Q320 chimeric brain, illustrating the plane of the sections shown in (**l–r**). **l–r** Dorso-ventral section series across the eminential region and some targets of eminential migrations of Q278, showing lack of QCPN-labeled cells. Blue arrowheads indicate the ventricular borders of the graft. Scale bars in **a**, 250 μm, **b**, 5 mm, **c-i**, and **l-r** 150 μm

To rule out non-eminential neighboring parts of the diencephalon as the source of cells migrated to the telencephalon, we made chimeras in which rostral parts of the presumptive diencephalon (Hb, Th, eventually including non-eminential PTh) were selectively grafted, ideally without inciding on the PThE (*n* = 11). We show here representative cases Q320 and Q278 (Fig. 9). Case Q320, sacrificed at HH37 (11 d.i.o.), is representative of controls including a small part of non-eminential prethalamus. Consistently with the fate map (Fig. 9a, b), this chimera only contained quail-derived cells at the Hb (brown labelled cells; Fig. 9c), dorsal part of Th (Fig. 9d–h), and dorsocaudal part of non-eminential PTh (Fig. 9d, e). The sections of this brain also were processed for *Tbr1* in situ hybridization. The reaction was somewhat pale, but nevertheless it identifies a blue PThE devoid of quail cells (Fig. 9e–g), and none such cells appear at the recipient nuclei of the eminential migrations (e.g., CoS, HiC, EA, POA, EW; Fig. 9f–i). Case Q278 received a graft only affecting the habenula and neighboring rostral part of thalamus (Fig. 9j–m). The Tbr1 reaction was clearcut in this case, identifying the PThE (Fig. 9n, o) and some of the targets of its migrations (Fig. 9n–r), all devoid of quail cells. These results jointly allow us concluding that the non-eminential PTh, Th and Hb do not participate in the source of eminential cells that migrate into the lateral hypothalamus and telencephalon.

## Discussion

We previously studied descriptively eminential tangential migrations, using their characteristic Tbr1 expression property (Alonso et al. 2020). The earliest eminential migrations presently observed experimentally correspond to mixed *juxtapeduncular* and *peripeduncular streams*, which can be distinguished incipiently at stages HH25-26. At more advanced stages (HH29-30), once the cerebral peduncle enlarges, these initial streams separate into an intermediate *juxtapeduncular component* (deep to the peduncle) and a subpial (marginal) *peripeduncular component*. The third eminential migratory component, *the eminentio-septal stream*, appears separately around stage HH29; it advances periventricularly rostralward under the interventricular foramen, to enter finally the septo-commissural area behind the anterior commissure. The peripeduncular, juxtapeduncular and eminentio-septal streams provide cells to different sites within the hypothalamic mantle layer, the caudal subpallium and the septal area, fully corroborating our previous descriptive conclusions (Alonso et al. 2020).

We had pointed out that the eminential derivatives occupy quite distinct adult recipient locations (peduncular hypothalamus, preoptic area, extended amygdala, diagonal band nuclei, posterior commissural septum and anterior commissure nucleus). They uniformly share habenulopetal connections (Nauta 1974; Herkenham and Nauta 1977; Swanson and Cowan 1979; Alonso et al. 1986; Otsu et al. 2018; Watanabe et al. 2018) and glutamatergic phenotypic properties (Abellán and Medina 2009; Abellán et al. 2010a,b; Puelles et al. 2012a, b; Wallace et al. 2017; Otsu et al. 2018; Watanabe et al. 2018; Agostineli et al. 2019). It is of interest that some centers receiving tangential migrations from the PThE, such as HiC, TS, MPO, MnPO, and DB, also independently receive additional migrated components from the pallial territory. Our present experimental results thus indicate conclusively that many *Tbr1*-positive glutamatergic neurons lying in the preopto-diagonal subpallium and superficial parts of the hypothalamic paraventricular area (plus associated parts of the lateral hypothalamus, including entopeduncular cells) derive from the PThE. The latter accordingly represents an important extratelencephalic source of tangentially migrated forebrain neurons, analogous in its relative topology next to the chorioidal roof plate and the migratory behaviour of most of its derivatives to the rhombic lip phenomenon in the hindbrain. We also conjectured that there might exist as yet unidentified eminential histogenetic compartments where eminential neuronal subpopulations originate that are destined by their subtly differential molecular profiles to entering different streams leading to distinct migratory targets and perhaps showing also medio-laterally differential habenular projection targets. See discussion of this point below.

Remarkably, our present studies did not detect in chick embryos any of the eminential tangential migrations described in the mouse which target *pallial territories,* such as the accessory olfactory bulb or the NLOT nucleus, or those equivalent to the eminential subpopulation of Cajal-Retzius cells that invades the mouse neocortex (see Puelles et al. 2000; Puelles 2011; Soriano and Del Rio 2005; Cabrera-Socorro et al. 2007; García-López et al. 2008; Abellán and Medina 2008, 2009; Abellán et al. 2010b; Medina and Abellán 2012; Huilgol et al. 2013; Ruiz-Reig and Studer 2017; Ruiz-Reig et al. 2017). The chicken eminential derivatives thus seem to respect the telencephalic pallial domains, in what represents a significant evolutionary difference with regard to the mammalian PThE counterparts.

### Technical considerations on the quail/chick chimeras

The quail/chick interspecific (chimeric) embryonic brain grafting technique (reviews in Le Douarin 1993, Alvarado-Mallart 2000; see also Guthrie 2008; Streit and Stern 2014) is a well-established, stable and specific method for tracing neuronal migrated cells, in fact all sorts of neuroepithelial derivatives (neurons, chorioidal tissue, glia cells). Avian embryos are easily accessible and handled. Embryonic homotopic neural grafts need to be combined with good fate maps performed at different early stages, which aid designing the experiments, placing the grafts, and ulterior interpretation of the experiments. Cells produced within the grafted territory can be traced from very early stages of development up to the adult. Whenever there is lack of radial coherence between the locus of quail-labelled grafted neuroepithelium (ependym) and quail cells in the mantle layer, evidence clearly arises of a tangential migration process, which may be followed with increasing survival times to its final destination. Even complex migratory routes with trajectories in three dimensions, which are difficult to visualize in thick brain slices, can be traced across series of sections, as occurred in the present case with the migrations that arise from the PThE. In addition, testing of migration hypotheses on whole embryos as opposed to brain slices, as occurs with the quail/chick grafting technique, avoids loss of the integrity of the neural structure, i.e., loss of signals which may be crucial for the migratory mechanism (Borrell and Marin 2006).

In this report we aimed to study eminential neurons known to selectively express *Tbr1* at early and late stages (Puelles et al. 2000; Alonso et al. 2020). The observed changing expression pattern of this marker over several embryonic stages spreads apparently from the PThE into hypothalamus, septum, preoptic area and other parts of subpallial telencephalon. This led us previously to the hypothesis that eminential neurons participate in three distinct streams of tangential migration coursing rostralward into hypothalamus and telencephalon through the subpial, intermediate and periventricular strata of the mantle layer, respectively (peripeduncular, juxtapeduncular and eminentio-septal streams of Alonso et al. 2020). The present quail/chick grafting experiments, abundantly cross-correlated with *Tbr1* expression, clearly corroborate this hypothesis.

### Comparison between the expression pattern of *Tbr1* and the quail cell migration pattern found in chimeric brains.

Our experiments with quail/chick chimeras (grafting small or large quail PThE pieces into chicken host PThE) indeed showed after appropriate survival times that numerous quail eminential cells migrate into the host hypothalamus (LH, EDP, SO), subpallium (Dg, LPO, POA, EA) and commissural septum (CoS, AC, TS, HIC). These tangential migrations clearly occur within the previously defined *Tbr1*-positive cell streams. The whole set of results accordingly corroborates experimentally our earlier interpretation of the *Tbr1*-positive PThE migratory streams (Alonso et al. 2020).

Our longitudinal descriptive results suggested that eminential tangential migration begins approximately between stage HH24 (4 days of incubation) and stage HH26 (5 days of incubation; Alonso et al. 2020 observed a distinct *Tbr1*-positive mantle at HH25-26, implying that eminential neurogenesis probably started some hours earlier, in agreement with general autoradiographic analyses of chicken diencephalic neurogenesis; Crossland and Uchwat 1982). It starts with the *juxtapeduncular migratory stream*, which advances precociously into the lateral hypothalamus and local incipient peduncle. One of our short-survival cases, which had a dorsal eminential graft, showed a group of *Tbr1*-positive juxtapeduncular migrating cells which lacked quail labelling (Fig. 2). We interpret this result as indicating that the juxtapeduncular migration is initiated by derivatives of the unlabelled ventral PThE. There is a ventrodorsal neurogenetic and histogenetic gradient across the alar plate of the avian diencephalon (Crossland and Uchwat 1983; Guillén-Pérez 1991); the quail-derived dorsal PThE probably was relatively delayed in its neurogenetic and migratory progress, and did not participate yet in the incipient migrating stream proceeding already at the ventral PThE. Such an overall maturation gradient of the developing diencephalon was also described using autoradiographic techniques in mammals (Angevine 1970; Altman and Bayer 1988), as well as by the analysis of acetylcholinesterase (AChE) expressing postmitotic neurons in chick embryos (Puelles et al. 1987). The earliest juxtapeduncular migratory cells thus probably originate from the ventral PThE, cross the hypothalamo-diencephalic boundary and enter the outer stratum of the neighboring hypothalamic paraventricular area at a stage (4–5 days in ovo) in which the peduncle is still poorly developed. Interestingly, the migrating juxtapeduncular cells do not penetrate the hypothalamic periventricular stratum, which is occupied by the paraventricular nucleus. Migrated eminential cells thus first accumulate deep to the peduncle (paraventricular lateral hypothalamus population) and partly inside the peduncle (prospective entopeduncular elements).

As development progresses, the superficial stratum of the peduncular hypothalamus is increasingly occupied by massive numbers of peduncular fibers, and the peduncle visibly represents a physical barrier to the intermediate migration. This obstacle possibly forces the subsequent eminential migratory flow (including probably more dorsal eminential cells coursing superficially within the PThE) to surround the peduncle subpially before proceeding rostralwards, thus originating the *peripeduncular migratory stream*. Young neurons from more dorsal eminential regions apparently start to migrate out of the PThE at stage HH28, as illustrated by some dorsal eminential grafts (e.g., case Q292). Such cases show the peripeduncular stream incipiently surrounding the subpial side of the peduncle, while the more advanced juxtapeduncular stream also presents numerous labelled cells, mainly in the LH. These results suggest that cells originated from ventral PThE largely follow the juxtapeduncular route into the hypothalamus and preoptic area, whereas dorsal PThE cells essentially enter the peripeduncular route targeting the basal telencephalon. As shown by cases fixed at stage HH34 (e.g., Q303) and stage HH35 (e.g., Q294), these two migratory contingents progressively reach and occupy their distal hypothalamo-preoptic (LH, LPO, MPO, MnPO, POI, and EPD) and subpallial (LPO, POI, Dg, BD, SO, and EA) targets at about 10 days in ovo.

Experimental data also reveal that the deep eminentio-septal stream is slightly retarded in its initial appearance when compared with the juxta- and peripeduncular ones. At stage HH28, only a handful of migrating eminential cells were found next to the ventricular zone of the hypothalamic paraventricular area (*Tbr1*-positive and quail-derived). Later, more abundant periventricular eminentio-septal migratory cells progressively move rostralwards under the interventricular foramen and along the telencephalic stalk (i.e., they cross longitudinally the peduncular alar hypothalamus—corresponding to the hp1 prosomere- and enter the terminal alar hypothalamus, or hp2 prosomere; Puelles et al. 2012a; Puelles and Rubenstein 2015). Eventually they reach the commissural septum (roof plate), after passing through the periventricular stratum behind the anterior commissure. Once inside the septum, the eminentio-septal stream arches caudalwards to reach the neighborhood of the hippocampal commissure (held to lie within hp1; Puelles et al. 2012a; Puelles and Rubenstein 2015), leaving derivatives along the whole septal course. At stage HH36, experimentally labelled eminential cells populate all the commissural septal nuclei: the anterior commissure nucleus (AC), the triangular septal nucleus (TS), the primordium of the commissural septal nucleus (CoS), and the hippocampal commissure nucleus (HiC). At this last locus (HiC) quail-labelled eminential cells apparently intermix with QCPN-negative *Tbr1*-positive pallial septo-commissural migrated counterparts originated from the medial pallium; these pallial streams, first described by Alonso et al (2020), never appear quail-labelled in our material. Interestingly, chimeras with small grafts restricted to the dorsalmost PThE labelled preferentially the eminentio-septal stream (e.g., case M25).

### Temporal vs. spatial origin of cell migrations from the PThE

In our previous longitudinal analysis of *Tbr1*-expressing cell migrations exiting from the PThE (Alonso et al. 2020) we suggested a possible subdivision of the eminential territory in positionally distinct compartments originating either the eminentio-septal stream, or the juxtapeduncular and peripeduncular streams associated to the peduncle. The evidence cited and discussed on that occasion referred to the reported differential connection pattern of various supposedly similarly migrated mammalian grisea (homologous with the present avian counterparts in the three streams) with the mammalian medial and lateral habenula. Eminential migrated derivatives projecting to the earlier born lateral habenula seemed to predominate in the juxta- and peripeduncular streams, whereas septocommissural elements studied both in mammals and lizards tended to project to the medial habenula, which has a retarded neurogenesis (see references on this point in Alonso et al. 2020). Remarkably, all migrated eminential derivatives project to the habenula, as does the non-migrated bed nucleus of the stria medullaris, the only remnant of the PThE in adult rodents (shown in Puelles et al. 2020).

In the present study we noticed additionally a subtle asynchrony between the three streams, i.e., a gradiental delay of the begin of the three migratory streams (first the juxtapeduncular stream appeared, next came the peripeduncular stream, and last the eminentio-septal stream). Small differences among our experiments suggested that the early juxtapeduncular contingents are generated ventrally (and possibly earlier) relative to the subsequently differentiated peripeduncular contingents, though our experiments were not sufficiently discriminative to demonstrate this point. Other inconclusive data suggested that the deep, retarded eminentio-septal stream was mainly affected in small dorsal grafts. This pattern points to a plausible temporal neurogenetic gradient across discrete eminential sources of the studied three migratory streams distributed along the ventro-dorsal dimension of the PThE primordium. More experimental work is needed to establish this point definitively.

Such a gradient may be related causally to both steplike histogenetic emergence of the three distinct migratory streams and a progressive latero-medial difference in the resulting habenular projections. The habenula itself is known to be patterned in its neurogenetic pattern, so that in both rodents and the chick lateral habenular cells are born first (stages HH21-28 or E3-E5.5 in chick; Guillén-Pérez 1991), whereas medial habenular neurons—with relatively more dorsal origins, next to the chorioidal tela- are born afterwards (stages HH28-30 or E5.5-E7 in chick; Guillén-Pérez 1991; Crossland and Uchwat 1982). This pattern suggests that a tiered ventrodorsal distribution of differential connective and migratory properties within the PThE may originate in a temporal ventrodorsal neurogenetic gradient, which may incide via differential postmitotic molecular properties on the beginning, detailed course, duration and final derivatives of each of the three migratory streams. We think it is simpler to assume that all postmitotic eminential cells first extend their axons into the habenular region (in ventrodorsal sequence), and perhaps even form incipient synaptic connections there, before the cell bodies start to exit the eminential progenitor area rostralward through the corresponding stream.

### Genetic heterogeneity across the prethalamic eminence

Various genes are expressed combinatorially in both the mouse and chick PThE. These include the transcription factors *Bmp4*, *Bmp6*, *Wnt3a*, *Wnt7b*, *Wnt8b*, *Wnt9a*, *Lef1*, *Sfrp2*, *Mkp3*, *Axin*, *p73*, *Grm1*, *Emx2*, *Ap2α* (*Tfap2a*), *Gdf10*, *Pax6, Neurog2*, *Tbr2*, *Calb2*, *Lhx1*, *Lhx5*, *Lhx1*, *vGlut2*, and *Tbr1* (Bulfone et al. 1995, 1999; Puelles et al. 2000; Crossley et al. 2001; Garda et al. 2002; Gimeno et al. 2003; Cabrera-Socorro et al. 2007; Vieira et al. 2009; Abellán et al. 2010b; Huigol et al. 2013; Adutwum-Ofosu et al. 2016; Ruiz-Reig and Studer 2017; Ruiz-Reig et al. 2017). Other characteristic markers are the Lot protein and the secreted morphogens FGF8 and FGF15 (Crossley et al. 2001; Gimeno et al. 2003; Ruiz-Reig et al. 2017; 2018). Genoarchitectonic analysis of the PThE is so far best documented in mouse embryos.

We are interested here in following the thread of apparent differential patterning and histogenesis along the ventrodorsal dimension of the PThE. There are practically no relevant data in the chick, but we have informative contributions on molecular compartmentation of the embryonic mouse PThE territory in the work of Adutwum-Ofosu et al. (2016) and Ruiz-Reig et al. (2017). Unfortunately, these authors continued using the obsolete classic notion of “thalamic eminence” (originated in historic times in which prethalamus was not distinguished from thalamus; now we know that the eminence is strictly a prethalamic component, sitting on diencephalic prosomere 3, rather than on prosomere 2; Puelles 2018, 2019; Alonso et al. 2020; this new scenario should not be disregarded). The cited two groups of authors distinguished topographically in standard embryonic brain cross-sections (which produce horizontal sections through the diencephalic prosomeres) three eminential portions: a *medial* part, which protrudes into the 3rd ventricle (their MTE), a *lateral* part, which extends the MTE into the medial aspect of the telencephalon, and lies behind and above the interventricular foramen (LTE), and an eminential *caudal hem* (C-hem), which receives a taenial insertion of the telencephalic chorioidal fissure (note Adutwum-Ofosu et al. 2016 did not use the C-hem term, though they did distinguish this area as a separate eminential part). The telencephalic *dorsal hem* (D-hem) distinguished by Ruiz-Reig et al. (2017) receives the other taenial fissural insertion, and represents the fimbrial or hippocampal counterpart of the C-hem. It is otherwise well known as the “cortical hem” in much recent work on cortical patterning. It may be deduced that the D-hem is telencephalic and cortical, whereas the C-hem is diencephalic and prethalamic eminential, so that their mutual topological relationship is not dorsoventral, as the cross-sectional topography would suggest, but rostrocaudal (reviews in Puelles 2011, 2019).

The three topographic eminential subdivisions of Adutwum-Ofosu et al. (2016) and Ruiz-Reig et al. (2017) can be translated into a embryologically and topologically more relevant ventro-dorsal one, resulting from the prosomeric notion of the PThE as an hyperdorsal progenitor area of the prethalamic alar plate, placed next to the local roof plate. In this concept the MTE would be ventralmost and the C-hem dorsalmost (Puelles et al. 2012a, 2020; Puelles and Rubenstein 2015). Indeed, the MTE represents the topologically *ventral PThE* part, which is connected to the remaining central and subcentral alar prethalamus derivatives (Puelles et al. 2020). The LTE corresponds to an *intermediate PThE* territory (in topographical relation with the interventricular foramen; we might also refer to this part as the ‘foraminal part of the eminence’). The C-hem is the topologically *dorsalmost PThE* part of the complex, since it contacts the chorioidal roof plate. It typically appears incorporated by evagination to the caudomedial telencephalic wall, and is limited by the chorioidal fissure. We can accordingly speak more appropriately of topologically *ventral, intermediate and dorsal PThE parts*. This original ventro-dorsal sequence is deformed during forebrain morphogenesis, so that in conventional coronal sections or cross-sections the three parts appear in a ‘medio-lateral’ MTE-LTE-C-hem topography, as observed by Adutwum-Ofosu et al. (2016) and Ruiz-Reig et al. (2017).

We insist on emphasizing the topological concept (ventral-intermediate-dorsal eminential parts) because of its clearcut relevance for causal patterning analysis, while the topographic medio-lateral schema is misleading in this respect. It largely reflects complex irrelevant morphogenetic epiphenomena (growth of the hemisphere relative to the neighboring prethalamus), and not intrinsic developmental phenomena of the area of interest. Indeed, due to the preference given conventionally to said post-morphogenetic topographic arrangement of eminential parts, few neurobiologists are aware of the fact that the fissural chorioidal tissue attached to the C-hem eminential area corresponds to the prethalamic roof plate (see Puelles 2019), which identifies the C-hem as a source of *dorsalizing* morphogenetic effects for the whole prethalamus.

Adutwum-Ofosu et al. (2016) examined in mouse embryos the differential eminential distribution of a number of gene markers representing the theoretically dorsalizing Wnt, Fgf and BMP families of morphogens, using the topographic schema of the PThE. Their results highlight that between E11.5 and E13.5 the MTE (topological ventral part of the eminence) is strongly characterized by *Wnt7b* signal (plus transient *Wnt7a* at E12.5), jointly with the Wnt negative modulator *Sfrp2*, and *Mkp3*. The LTE (intermediate or foraminal part of the eminence) also expresses *Wnt7b* (less strongly), in this case jointly with *Wnt8b*, *Sfrp2,* the morphogen *Fgf8,* and its downstream target *Mkp3*. *Grm1* expression and Lot protein were also reported as being selectively present at the LTE (Ruiz-Reig et al. 2017). The MTE is thus distinguished from the LTE mainly by the absence of *Fgf8*.

Finally, the C-hem domain (dorsalmost part of the eminence) lacks *Wnt7b* altogether but expresses overlapping *Wnt8b* and *Wnt9a* and transient *Wnt3a* (only at E12.5), jointly with *Bmp6* and the downstream gene *Axin2*. In addition, *p73* was reported selectively therein by Ruiz-Reig et al. (2017). Moreover, the C-hem (dorsalmost part of the PThE) also expresses *Lef1*, *Emx2* and *Tfap2a* (Garda et al. 2002; Adutwum-Ofosu et al. 2016; Ruiz-Reig et al. 2017). This subdomain is negative for *Wnt7b*, *Sfrp2*, *Mkp3*, *Grm1*, *Neurog2*, and *Fgf15*. Some authors relate it to a subpopulation of corticopetal Cajal-Retzius neurons (migration discussed in Alonso et al. 2020; Tissir et al. 2009; Miquelajáuregui et al. 2010; Puelles 2011). Interestingly, the fimbrial (hippocampal) D-hem domain of the medial telencephalic wall also shows selective *Wnt9a, Axin2* and *Bmp6* expression, but lacks *Wnt8b* signal (Adutwum-Ofosu et al. 2016), thus showing a qualitative difference with the C-hem subdomain. *Bmp6* is expressed strongly at the chorioidal fissure roof plate.

This set of markers accordingly reveals that the topologically ventro-dorsal molecular eminential subdivision starts at the ventral part of the eminence (MTE) with *Wnt7b* (plus *Wnt3a*), changes in the intermediate eminential region (LTE) to an overlap of *Wnt7b* and *Wnt8b* with *Grm1*, *Lot*, and *Fgf8*, and adopts dorsally (C-hem) the combination of *Wnt8b* and *Wnt9a*. *Sfrp2* is shared by the two lower parts, while *Bmp6*, a roof marker, is shared by the two upper parts. A combinatorial Wnt code with other auxiliary markers is thus apparent, which may participate in specifying differentially the identity of each of the three postulated progenitor subareas across the prethalamic eminence, with possible consequences in both connection targets and tangential migration pathways and final sites of the derived eminential subpopulations. We pointed out in the previous work (Alonso et al. 2020) that the juxtapeduncular, peripeduncular and eminentio-septal migratory streams are conserved evolutionarily.

### Differences between mouse and chick eminential tangential migrations.

In general, the hypothalamic and telencephalic (preoptic, basal and septocommissural) eminential migratory targets described by us in the chick (Alonso et al. 2020; present results) also can be identified in the mouse (Nauta 1974; Herkenham and Nauta 1977; Carter and Fibiger 1978; Parent 1979; Parent et al. 1981; van der Kooy and Carter 1981; Parent and De Bellefeuille 1982; Parent et al. 1984; Parent 1986; Namboodiri et al. 2016; Zahm and Root 2017; Wallace et al. 2017; Watanabe et al. 2018; discussion in Alonso et al. 2020; see also Puelles et al. 2020). In contrast, additional mouse eminential cell populations have been found to migrate tangentially to diverse targets in the telencephalic pallium, notably the pyriform cortex (Lot guidepost cells) and pAOB (Ruiz-Reig and Studer 2017; Puelles 2011). Corroborating conclusions already advanced in our previous descriptive analysis (Alonso et al. 2020), we did not detect any palliopetal phenomena in our present experimental chicken material. Remarkably, many of the reported mammalian pallial targets are related to the olfactory system (pyriform cortex, pAOB and NLOT), a system that is poorly developed and is perhaps also somewhat dispersed in birds (Puelles 2017). Indeed, whereas the mouse lateral olfactory tract (lot) appears strictly localized to the subpial stratum of the ventral pallium domain (Puelles 2014; Puelles et al. 2016), in the chick the lot fibers are less abundant in the pallium, and many of them also spread widely within the subpial stratum of the subpallial olfactory tuberculum, meriting the appellative ‘ventral olfactory tract’ (Puelles et al. 2007, 2019). Moreover, birds lack an accessory olfactory bulb. As commented in Alonso et al. (2020), it is possible that eminential sign-post neurons or other cells emitting guidance signals may not be available to allow the olfactory bulb efferent axons to fasciculate as in mammals. Unfortunately, we still lack proper molecular mapping of such potential palliopetal cell populations in the chick and other birds. Some avian olfactory fibers nevertheless reach finally the amygdalar subpial domain as well as the postulated entorhinal cortex homolog (Puelles et al. 2007, 2019). As regards the alleged eminential Cajal-Retzius cell population (see critical comments on previous use of the p73 marker in this regard in Alonso et al. 2020), birds generally are noted for a poorly developed system of Cajal-Retzius cells (Tissir et al. 2002; Abellán et al. 2010a).

One conjecture which may merit future exploration is that the substantial avian eminential migration which targets the *extended subpallial amygdala* possibly represents cells equivalent in other respects to those invading the olfactory pallium in mammals; these cells might not be able to traverse the pallio-subpallial boundary, thus remaining stuck in the subpallium (we previously noted their *Tbr1*-positive and glutamatergic phenotype, inconsistent with a subpallial origin; García-López et al. 2008; Abellán et al. 2010b; Bupesh et al. 2011; Alonso et al. 2020).

### Possible mechanisms involved in PThE migrations

One question that remains to be approached is why most cells produced at the mouse and chicken prethalamic eminence migrate rostralwards, in contrast with those of other parts of the prethalamus and neighboring thalamus/habenula areas, which show comparatively a rather static behaviour, as our experimental controls demonstrate (Fig. 9; Table 1). The uniform general rostral direction of migration (coinciding with uniform caudally oriented habenular connections), and accompanying timing differences in the stream used and the targets reached, suggests to us that a repellent cause (or causes) should be searched locally, at the PThE itself, or close by, as we already suggested in Alonso et al. (2020). It would seem difficult to orchestrate a sufficient multiplicity of correlative attractor effects, though *Fgf8* signal present at the commissural septum might be significant, due to evidence that it may attract glutamatergic neurons (Pombero et al. 2011). Likewise, the POA broadly expresses SHH protein theoretically capable of an attractive role at stages previous and coincident with eminential migrations (Angot et al. 2008; Bardet et al. 2010; Abellán and Medina 2009).

The PThE domain itself (or one of its three parts) perhaps generates signals repellent for eminential derivatives, which as a result tend to sort out of that environment in the most permissive direction (rostralwards). As commented in the Introduction, this hypothesis recalls what is known to happens with rhombic lip cells of the hindbrain, which also are born at the dorsal border of the local alar plate. In support of this idea, there is expression of several dorsal diffusing morphogens (Wnts, *Bmp6* and *Fgf8*) at the dorsalmost part of prosomere 3, i.e., at the PThE. Moreover, the transcripts of both *p73* and *Grm1*, likewise expressed in this area, have been related functionally to the promotion of cell migration (Sablina et al. 2003; Cabrera-Socorro et al. 2007; Tissir et al. 2009; Landré et al. 2016; Isola et al. 2018; Ruiz-Reig et al. 2017).

In that case, one would expect that eminential cells might exit unspecifically in several directions, or, at least, both rostralwards and ventralwards. However, migratory capability may be somehow conditioned to previous axonal connection with the habenula (and retrograde transport of some signal from there?). Secondary dependence of migration on having an axonal interaction with the habenula might restrict the direction of migration to the hab-axonal rostral pole of the eminential cells. Alternatively, (or in addition), a repellent signal may be generated by the habenular area itself which diffuses directly, or is transmitted retrogradely via the eminential axons, to the eminential cell bodies. Another known potential local source of diffusing signals is the transverse prethalamo-thalamic boundary, the zona limitans, whose dorsal tip separates the PThE from the habenular domain. Complementarily, it stands to reason that each of the migratory targets probably offers some peculiar local conditions which favour stopping the migrating behavior and local integration of some components of the streams, perhaps acting selectively on some cellular subtypes.

## Abbreviations

ac: Anterior commissure
AC: Nucleus of anterior commissure
AH: Amygdalohippocampal area
ATn: Amygdalar taenial nucleus
cht: Chorioidal tela
csm: Cortico-septo-mesencephalic tract
CoS: Commissural septal nucleus
CoSM: Commissural septal nucleus medial part
CoSL: Commissural septal nucleus lateral part
DB: Diagonal band nuclei
DPall: Dorsal pallium
Dg: Diagonal band area
EA: Subpallial extended amygdala
ech: Eminential chorioidal tela
EPD: Dorsal entopeduncular nucleus
ESA: Eminentio-septal area
EW: Eminential wings
fich: Telencephalic fimbrial chorioidal tela
Hb: Habenula
HDB: Horizontal limb of the diagonal area
Hi: Hippocampus
hic: Hippocampal commissure
HiC: Hippocampal commissure nucleus
hp1: Hypothalamic prosomere 1
ivf: Interventricular foramen
IIIv: Third ventricle
LH: Lateral hypothalamus
LPall: Lateral pallium
LPO: Lateral preoptic area
lv: Lateral ventricle
MPall: Medial pallium
MPO: Medial preoptic nucleus
MnPO: Paramedian preoptic nucleus
MPall: Medial pallium
oc: Optic chiasm
ot: Olfactory tubercle
p2: Prosomere 2
p3: Prosomere 3
Pa: Paraventricular nucleus
Pal: Pallidus
Pall: Pallium
PallA: Pallial amygdala
pe: Cerebral peduncle
POA: Preoptic area
POH: Preoptohypothalamic area
POI: Preoptic Island
PT: Pretectum
PTh: Prethalamus
PThE: Prethalamic eminence
Re: Retina
Se: Septum
sm: Stria medullaris tract
SO: Supraoptic nucleus
Spall: Subpallium
St: Striatum
svz: Subventricular zone
Th: Thalamus
thch: Thalamic chorioidal tela
TS: Triangular septal nucleus
TP: Temporal pole
VDB: Vertical limb of the diagonal area
VPall: Ventral pallium
vz: Ventricular zone
zli: Zona limitans interthalamica
